# Aryl‐Modified Pentamethyl Cyanine Dyes at the C2’ Position: A Tunable Platform for Activatable Photosensitizers

**DOI:** 10.1002/advs.202305761

**Published:** 2023-12-08

**Authors:** Fuping Han, Syed Ali Abbas Abedi, Shan He, Han Zhang, Saran Long, Xiao Zhou, Supphachok Chanmungkalakul, He Ma, Wen Sun, Xiaogang Liu, Jianjun Du, Jiangli Fan, Xiaojun Peng

**Affiliations:** ^1^ State Key Laboratory of Fine Chemicals Frontiers Science Center for Smart Materials Dalian University of Technology Dalian 116024 China; ^2^ Fluorescence Research Group Singapore University of Technology and Design Singapore 487372 Singapore; ^3^ Department of Chemistry Hong Kong Branch of Chinese National Engineering Research Center for Tissue Restoration and Reconstruction and Institute for Advanced Study The Hong Kong University of Science and Technology Clear Water Bay Kowloon Hong Kong China; ^4^ Ningbo Institute of Dalian University of Technology Dalian University of Technology 26 Yucai Road, Jiangbei District Ningbo 315016 China

**Keywords:** cyanine, fluorophore engineering, photodynamic therapy, photo‐induced electron transfers, twisted intramolecular charge transfers

## Abstract

Pentamethyl cyanine dyes are promising fluorophores for fluorescence sensing and imaging. However, advanced biomedical applications require enhanced control of their excited‐state properties. Herein, a synthetic approach for attaching aryl substituents at the C2’ position of the thio‐pentamethine cyanine (TCy5) dye structure is reported for the first time. C2’‐aryl substitution enables the regulation of both the twisted intramolecular charge transfer (TICT) and photoinduced electron transfer (PET) mechanisms to be regulated in the excited state. Modulation of these mechanisms allows the design of a nitroreductase‐activatable TCy5 fluorophore for hypoxic tumor photodynamic therapy and fluorescence imaging. These C2’‐aryl TCy5 dyes provide a tunable platform for engineering cyanine dyes tailored to sophisticated biological applications, such as photodynamic therapy.

## Graphical abstract

This work reports a promising pentamethyl cyanine platform using C2’‐aryl substitutions. It is found that electron‐withdrawing groups enhanced twisted intramolecular charge transfer (TICT), resulting in efficient fluorescence quenching. The electron‐donating groups make the TICT tendency weaken significantly, while photoinduced electron transfer is enhanced. This change improves both the fluorescence quantum yield and the intersystem crossing (ISC) efficiency.

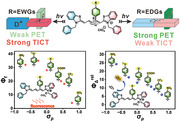

## 1 Introduction

Cyanine, originally proposed as a classic imaging chromophore, is favorable among existing dyes because it combines high extinction coefficients (>10^5^ M^−1^ cm^−1^), good biocompatibilities, and high water solubilities.^[^ ^1^ ^]^ Within the cyanine family, the pentamethyl cyanine dye (Cy5) exhibits near‐infrared absorption and emission (>650 nm), in addition to an excellent photostability, thereby rendering it one of the best candidates for fluorescence imaging, sensing, and photodynamic therapy (PDT).^[^ ^2^ ^]^ However, with the increase in demand for advanced functions and applications, the need for Cy5 structures that can regulate the release of the excited‐state energy becomes increasingly significant.^[^ ^3^ ^]^ For example, high fluorescence quantum yields (*Φ*_f_) and intersystem crossing (ISC) efficiencies are both crucial requirements for fluorescence image‐guided PDT, while these processes inherently compete with one another.^[^ ^4^ ^]^ Therefore, there is an urgent requirement to explore new molecular designs for regulating the various photophysical mechanisms and achieving the desired performances.

Many strategies to improve ISC have been proposed for developing efficient near‐infrared photosensitizers.^[^ ^5^ ^]^ However, the strategies for modifying Cy5 focus on the meso‐position of the conjugate chains, owing to the ease of synthesis.^[^ ^6^ ^]^ For example, anthracene has been introduced as an electron donor at the meso‐position, which activates the spin‐orbit charge transfer ISC (SOCT‐ISC) via the occurrence of the photoinduced electron transfer (PET) and effectively generates long‐lived triplet excited states.^[^ ^7^ ^]^ Alternatively, the introduction of an electron‐withdrawing group (EWG) at the meso‐position of Cy5 can reduce the S_1_−T_1_ energy gap (Δ*E*_S−T_), which promotes the production of a significant amount of reactive oxygen species (ROS) for PDT through the ISC process.^[^ ^8^ ^]^ However, modification at the meso‐positions to enhance the ISC efficiency also hinders dissipation of the excited state energy through fluorescence.^[8a,9]^ Therefore, new mechanisms for the co‐regulation of these multiple processes must be developed to overcome such adverse effects.

To optimize the photophysical properties of Cy5 dyes, one attractive option is to attach substituents at other positions along the π‐bridge of the cyanine moiety and investigate the corresponding structure–property relationship.^[^ ^10^ ^]^ Notably, modifications at the C2’ aryl group could enhance ISC through PET while modulating fluorescence via twisted intramolecular charge transfer (TICT).^[^ ^11^ ^]^ By jointly engaging TICT and PET, this approach may overcome the usual competition between fluorescence and the formation of triplet states. It could simultaneously boost fluorescence and ISC. However, the reported synthetic routes of C2' substitution are complex and not universal,^[^11a^,^12^]^ limiting the development of new Cy5 derivatives with tailored properties.

**Figure 1:**
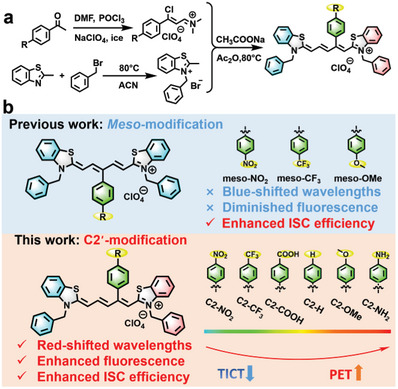
a) Schematic illustration of the synthesis of C2’‐modified TCy5 dyes. b) Comparison of the photophysical properties of the meso‐modified TCy5 dyes and the C2’‐modified TCy5 dyes.

As shown in **Figure** **1**a, we herein report a three‐step synthesis method using acetophenone to achieve C2’‐aryl substitution in thio‐pentamethine cyanine (TCy5) dyes C2‐R (R = NO_2_, CF_3_, COOH, H, OMe, NH_2_). Varying para‐substituents on the aryl group effectively tune TCy5 photophysics. The *Φ*_f_ and the generation of ROS simultaneously increased with the electron‐donating strength of the para‐substituents in the aryl group (Figure 1b). Quantum calculations show that as the electron‐donating ability of the aryl substituent rises, TICT weakens, improving *Φ*_f_, while PET enhances, facilitating ISC through SOCT‐ISC. Nitroreductase (NTR), overexpressed in hypoxic tumor cells, can convert nitro to amino efficiently.^[^ ^13^ ^]^ Therefore, an EWG‐modified C2‐NO_2_ is developed as a photosensitizer precursor with weak fluorescence and a low ROS production capacity in normal cells. In hypoxic tumor cells, C2‐NO_2_ was converted into C2‐NH_2_ to yield bright fluorescence and generate large amounts of ROS to kill the tumor cells. This structure‐based strategy should provide an effective guide for the development of advanced functional dyes.

## 2 Results and Discussion

### 2.1 Molecular Design and Synthesis

To obtain the C2’‐modified cyanine dyes, an intermediate condensate was prepared via the Vilsmeier–Haack reaction using acetophenone as a reagent. The C2’ aryl‐modified dye C2‐H is prepared by reacting the condensate with a quaternary ammonium salt via the Knoevenagel reaction (Schemes S1–S4, Supporting Information). This synthetic method involves the universal attachment of electron‐withdrawing groups (e. g. C2‐NO_2_, C2‐CF_3,_ and C2‐COOH) and electron‐donating groups (e. g. C2‐OMe and C2‐NH_2_) to C2‐H (Figure 1b). The substitution position of the aryl group was confirmed by the resulting crystal structure (Figure S1, Supporting Information). The conventional Cy5 derivatives TCy5‐H, meso‐OMe, meso‐CF_3,_ and meso‐NO_2_ were also prepared as reference compounds following synthetic methods reported in the literature.^[^8a^]^ The structures of all the above dyes are shown in Figure S2 (Supporting Information) and were all confirmed by ^1^H NMR, ^13^C NMR, and ESI‐HRMS (Figures S38–S61, Supporting Information).

### 2.2 Photophysical Characterization

**Figure 2:**
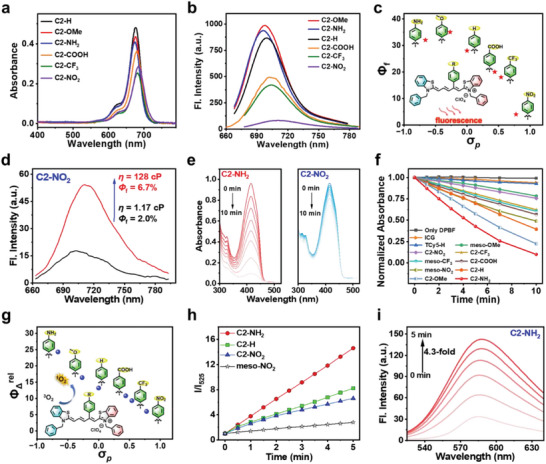
Evaluation of the photophysical properties of the cyanine dyes. a) Absorbance spectra of the various dyes in DCM (2 µM). b) Fluorescence spectra of the various dyes in DCM (2 µM). c) Relationship between the dye *Φ*_f_ of dyes and the para‐substituent constant (*σ*_p_). d) Fluorescence spectra of C2‐NO_2_ in 1, 5‐pentanediol (red), and ethanol (black) at 20 °C. e) DPBF degradation induced by C2‐NH_2_ and C2‐NO_2_ under 660 nm light irradiation (5 mW cm^−2^) for different durations. f) Normalized DPBF absorbance at 415 nm, demonstrating the degradation induced by different compounds under 660 nm light irradiation. g) Relationship between the *Φ*_Δ_^rel^ values of the dyes and *σ*_p_. h) Fluorescence response of DHR123 (10 µM) in the presence of aqueous solutions of C2‐NH_2_, C2‐H, meso‐NO_2_, and C2‐NO_2_ (5 µM) in aqueous solution under 660 nm light irradiation. i) Fluorescence response of DHE (15 µM) in the presence of aqueous solution of C2‐NH_2_ (5 µM) under 660 nm light irradiation.

Initially, the UV–vis–NIR absorption spectra of the compounds were recorded and analyzed, as shown in **Figure** **2**a and **Table** **1**. Consequently, it was found the peak absorption wavelengths (λ_abs_) of the C2’‐modified cyanine dyes were longer than that of the reference unsubstituted compound TCy5‐H. Among the various C2’‐modified cyanine dyes, the EWG‐modified dyes (C2‐NO_2_, C2‐CF_3,_ and C2‐COOH) displayed a bathochromic shift in comparison to the EDG‐modified dyes (C2‐OMe and C2‐NH_2_). In addition, the molar extinction coefficients of the C2’‐modified cyanine dyes were determined to be >10^5^ M^−1^ cm^−1^ (Table 1), thereby demonstrating their excellent photon‐absorbing capabilities. More importantly, the modification of the C2’‐position doesn't reduce the light stability of the dye, which is desirable for their potential use in PDT/ imaging applications (Figure S3, Supporting Information).

**Table 1:** Photophysical Properties of the Cy5‐Derivatives.

compd.^a)^	λ_abs_ ^b)^	λ_em_ ^c)^	*ε* ^d)^	*Φ*_f_ ^e)^	*Φ*_Δ_^rel^ ^f)^	τ_T_ ^h)^
C2‐NO_2_	685	710	14.4	4	6.0	9.8
C2‐CF_3_	682	703	12.7	18	9.3	‐ ^h)^
C2‐COOH	680	702	18.3	20	10.8	‐ ^h)^
C2‐H	676	700	24.1	28	13.2	‐ ^h)^
C2‐OMe	675	698	21.8	35	16.4	34.3
C2‐NH_2_	674	696	20.4	32	24.3	12.1
TCy5‐H	668	690	19.1	56	2.3	‐ ^h)^
meso‐NO_2_	664	684	12.0	23	11.0	‐ ^h)^
meso‐CF_3_	666	686	11.4	31	9.9	‐ ^h)^
meso‐OMe	673	697	9.5	64	4.4	‐ ^h)^

^a)^ All the properties were determined in dichloromethane;

^b)^ Maximum absorption wavelength (nm);

^c)^ Maximum emission wavelength (nm);

^d)^ Molar absorption coefficient, 10^4^ M^−1^ cm^−1^;

^e)^ Absolute fluorescence quantum yield;

^f)^ Relative singlet oxygen quantum yield with ICG as standard (*Φ*_Δ_^rel^ = 1);

^g)^ Intrinsic triplet excited‐state lifetime in microseconds (µs);

^h)^ Not applicable.

Subsequently, the fluorescence properties of the C2’‐modified cyanine dyes were evaluated. As shown in Figure 2b and Table 1, the λ_em_ values of the new dyes were higher than those of TCy5‐H, particularly in the case of the EWGs‐substituted fluorophores. Furthermore, the *Φ*_f_ of the C2′‐modified Cy5 dyes (4 and 35%, as measured in dichloromethane) was lower than that of TCy5‐H (56%). As the electron‐donating ability of the substituent increased, the quantum yields gradually increased from 4% (C2‐NO_2_) to 32% (C2‐NH_2_) and 35% (C2‐OMe). These results indicate that the *Φ*_f_ of the dye increases upon narrowing of the para‐substituent constant (*σ*_p_) of the aryl group (Figure 2c). These changes in *Φ*_f_ demonstrate that adjusting the push‐pull effects via substitution can effectively change the fluorescence intensity of the dye.

The fluorescent properties of the C2‐NO_2_ and C2‐NH_2_ dyes were subsequently examined in different solvents. It was found that the fluorescence intensities of both dyes were affected by the solvent polarity, wherein the fluorescence faded in highly polar solvents and gradually increased upon decreasing the solvent polarity (Figure S4, Supporting Information). The effects of the solvent viscosity were also examined using ethanol and 1,5‐pentanediol, which have the same polarity (i.e., ET^N^ = 0.65)^[^ ^14^ ^]^ but different viscosities (i.e., 1,5‐pentanediol > ethanol). As shown in Figure 2d, C2‐NO_2_ exhibited stronger fluorescence in the 1,5‐pentanediol (*Φ*_f_ = 6.7%) than in the ethanol (*Φ*_f_ = 2.0%), likely due to the higher‐viscosity solvents restricting bond rotation and thereby inhibiting TICT. Interestingly, as shown in Figure S5 (Supporting Information), although the fluorescence intensity of C2‐NH_2_ in the high viscosity 1, 5‐pentanediol (*Φ*_f_ = 25.1%) was enhanced to 1.9‐fold over that in ethanol (*Φ*_f_ = 12.9%), the enhancement ratio was less than that of C2‐NO_2_ (3.4‐fold). This difference indicates that C2‐NH_2_ is less affected by TICT.

The ROS generation ability is a critical indicator for verifying the ISC efficiency of a dye and evaluating its potential as a photosensitizer. Thus, 1,3‐diphenylisobenzofuran (DPBF) was used as an indicator to assess the ability of the different dyes to form singlet oxygen (^1^O_2_) in DCM under NIR irradiation (5 mW cm^−2^, 660 nm). As shown in Figure 2e,f and Figure S5, the absorbance of DPBF (at 415 nm) decreased in the presence of other dyes. Using ICG as a standard, we calculated the relative singlet oxygen quantum yield (*Φ*_Δ_^rel^) of each dye (Table 1 and Table S1, Supporting Information), confirming that the C2’‐modified cyanine dyes possessed higher *Φ*_Δ_^rel^ values than TCy5‐H. Moreover, the electron‐donating ability of the C2′ substituent was found to correlate positively with the value of *Φ*_Δ_^rel^, as demonstrated by the increase in *Φ*_Δ_^rel^ from 6.0 for C2‐NO_2_ to 24.3 for C2‐NH_2_. As shown in Figure 2g, the *Φ*_Δ_^rel^ of the photosensitizer increased (6.0 to 24.3) as the *σ*_p_ on the aryl group decreased (i.e., representing enhancement of the electron‐donating ability). As a result, the introduction of a strong EDG at the C2’‐position of TCy5 dramatically enhanced the ^1^O_2_ production. Notably, the *Φ*_Δ_^rel^ of C2‐NH_2_ surpassed even that of the meso‐modified dyes meso‐OMe (4.4) and meso‐NO_2_ (11.0), indicating that aryl modification at the C2’‐position could provide photosensitizers with exceptional performance. Fluorescence experiments using Singlet Oxygen Sensor Green as ^1^O_2_ probe confirmed that C2‐R dyes could generate singlet oxygen in an aqueous solvent (Figure S7, Supporting Information).

As another type of ROS, the superoxide anion (O_2_^•−^) is known to be effective in eliminating hypoxic tumor cells.^[^ ^15^ ^]^ To investigate this further, dihydrorhodamine 123 (DHR123) and dihydroethidium (DHE) were used as probes to study the O_2_^•−^ generation capacities of C2‐NO_2_, C2‐H, C2‐NH_2_, and meso‐NO_2_. In the first set of experiments, the dyes (5 µM) and DHR123 (10 µM) were dissolved in water and subjected to light irradiation (660 nm LED lamp), which led to a continuous increase in the fluorescence intensity at 525 nm (Figure 2h and Figure S8, Supporting Information). After 5 min of irradiation, the fluorescence intensity of the C2‐NH_2_ group increased 14.6‐fold, which was greater than the corresponding values obtained for C2‐NO_2_ (2.8‐fold) and meso‐NO_2_ (6.6‐fold). In the subsequent, the dyes (5 µM), DHE (15 µM), and ctDNA (100 µg mL^−1^) were mixed and irradiated at 660 nm using an LED lamp. Similarly, as shown in Figure 2i, the fluorescence intensity (570–600 nm) of the C2‐NH_2_ group increased 4.3‐fold, which was a greater increase than those of C2‐NO_2_ (2.8‐fold) and meso‐NO_2_ (3.0‐fold) (Figure S9, Supporting Information). Furthermore, the electron spin resonance (ESR) spectra of TEMPO and DMPO were measured to determine the type of ROS produced by C2‐NH_2_. The results indicated that only the irradiated group produced a significant signal (Figure S10, Supporting Information), and the obtained spectra confirmed the generation of both ^1^O_2_ and O_2_^•−^. In summary, C2‐NH_2_ can generate a variety of ROS through electron and energy transfer with O_2_.

### 2.3 Quantum Chemical Calculation

**Figure 3:**
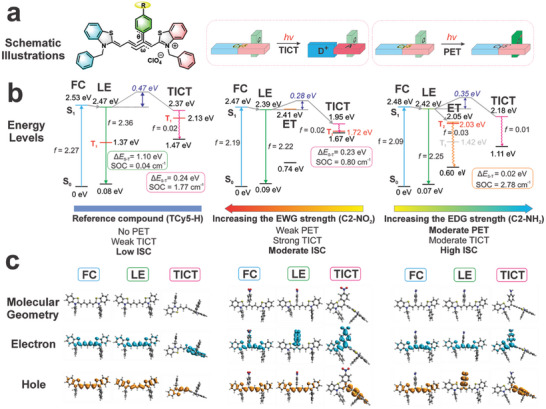
Results of quantum chemical calculations. a) Schematic illustrations of the TICT and PET mechanisms in the C2’ aryl‐modified thio‐pentamethine cyanine dyes. b) Energy levels and oscillator strengths f) of the key states involved during the inter‐system crossing of TCy5‐H (left panel), C2‐NO_2_ (the middle panel), and C2‐NH_2_ (the right panel) in DCM. The inset shows the Δ*E*_S−T_ and SOC values of the representative states. c) Optimized geometries, and the corresponding electron and hole distributions of representative states of TCy5‐H (left panel), C2‐NO_2_ (the middle panel), and C2‐NH_2_ (the right panel). FC: Franck Condon state; LE: adiabatically locally excited state; ET: adiabatically electron‐transfer state; TICT: twisted intramolecular charge transfer state.

Subsequently, quantum chemical calculations were performed to elucidate the photophysical characteristics of the C2’‐modified Cy5 derivatives. Cyanine dyes are prone to TICT, which results in complete charge separation and near‐zero oscillator strength, thereby rendering such compounds weak or non‐emissive.^[^ ^16^ ^]^ Moreover, PET can occur between the Cy5 framework and the aryl substituent connected to the polymethine bridge.^[^ ^17^ ^]^ The ensuing electron transfer (ET) state is also non‐emissive, negatively affecting the *Φ*_f_ of the fluorophores. Nevertheless, the ET state has been demonstrated to act as a crucial intermediate state for intersystem crossing.^[^ ^11^, ^18^ ^]^ Consequently, we considered both TICT and PET during the computational modeling (**Figure** **3**a).

The calculations revealed that the reference compound TCy5‐H exhibited low triplet formation yields but high *Φ*_f_. This can be attributed to the absence of a C2’‐substituent, which prevents PET from occurring in TCy5‐H. Furthermore, TCy5‐H faces a significant energy barrier (0.47 eV) and experiences weak driving energy (≈0.10 eV) to enter a TICT state (Figure S9, Supporting Information). Interestingly, as shown in Figure 3b, the energy gap between the singlet and triplet states (Δ*E*_S−T_) in the TICT state (0.24 eV) is significantly smaller than that in the planar locally excited (LE) state (1.10 eV), while the spin‐orbit coupling (SOC) in the TICT state (1.77 cm^−1^) is significantly larger than that of the LE state (0.04 cm^−1^). The small Δ*E*_S−T_ and large SOC values suggest that the TICT state serves as an effective pathway to facilitate ISC. However, owing to the weak tendency of TCy5‐H to undergo TICT, its triplet formation yield is low, whereas its *Φ*_f_ remains high.

The calculations also showed that attaching an electron‐withdrawing group to the C2’‐subsituend phenyl rings could moderately improve the ISC by enhancing the TICT tendency. PET is less stable than the LE state in C2‐NO_2_, rendering a low probability of entering the corresponding ET state. However, due to the asymmetrical molecular structure and enhanced push‐pull effect in the twisted conformation, the TICT tendency of C2‐NO_2_ was considerably amplified. As a result, the driving energy to access the TICT state increases to 0.44 eV in C2‐NO_2_ in DCM (c.f., 0.10 eV in TCy5‐H; Figure 3b). Simultaneously, the energy barrier to enter the TICT state is reduced to 0.28 eV in C2‐NO_2_ (c.f., 0.44 eV in TCy5‐H). The substantial population of the TICT state therefore significantly diminishes the *Φ*_f_ value of C2‐NO_2_ to only 4%. Fortunately, upon TICT rotations, Δ*E*_S−T_ decreases to 0.23 eV in the TICT state, while SOC is augmented to 0.80 cm^−1^ in the TICT state (Figure S12, Supporting Information). The enhanced TICT tendency in C2‐NO_2_ consequently enhances the *Φ*_Δ_^rel^ of C2‐NO_2_ value to 6.0 (c.f., 2.3 for the reference compound TCy5‐H).

As the electron‐donating strength of the aryl substituent was gradually increased, the TICT tendency diminished considerably. For instance, the energy barrier to access the TICT state in C2‐NH_2_ was found to rise to 0.35 eV, while the driving energy to enter the TICT state decreased to 0.24 eV. Simultaneously, PET became viable in C2‐NH_2_, and the corresponding ET state became 0.37 eV more stable than the LE state of C2‐NH_2_ (Figure S13, Supporting Information). Due to the substantial inhibition of TICT and the moderate enhancement of PET, the overall *Φ*_f_ increased to 32% in C2‐NH_2_, which is significantly higher than that of C2‐NO_2_ (i.e., 4%), yet still lower than that of the reference compound TCy5‐H (56%). The presence of the ET state not only minimized the Δ*E*_S−T_ value (to 0.02 eV) but also substantially amplified the SOC value (to 2.78 cm^−1^); improvements in both parameters significantly enhanced the triplet formation yields (Figure 3b). Due to the presence of this stable ET state, C2‐NH_2_ exhibited the highest triplet formation yield (*Φ*_Δ_^rel^ = 24.3). This efficient ISC is crucial for achieving a high photosensitizer performance.

The differences between PET and TICT were further examined to ensure an efficient ISC. It should be noted here that although the formation of both the ET and TICT states minimizes Δ*E*_S−T_ and enhances SOC, one key distinction between these two states is their optical gaps. As shown in Figure 3c, a small optical gap exists in the TICT state (i.e., 0.28 eV in C2‐NO_2_), because of twisting the fluorophore scaffold, substantially promoting internal conversion, and leading to significant nonradiative decay (the energy gap law). Such high nonradiative decay is detrimental to ISC. Consequently, PET (with a relatively large optical gap, i.e., 1.45 eV in C2‐NH_2_) is preferred over TICT for facilitating ISC. Interestingly, modification of the aryl substituents in C2’‐substituted Cy5 derivatives provides a convenient strategy for adjusting the TICT/PET tendency. It was found that as the substituent progressively transitioned from a strongly electron‐withdrawing group (e.g., –NO_2_) to a strongly electron‐donating group (e.g., –NH_2_), the TICT tendency decreased, whereas the PET tendency was enhanced (Figures S12–S15, Supporting Information). This switch improved the overall triplet formation yields (and the *Φ*_f_ simultaneously). Indeed, experimental measurements also demonstrated a strong correlation between the Hammett values of the aryl substituents and the triplet formation yields of these C2’‐substituted Cy5 derivatives. These computational data show that strong donors yield enhanced *Φ*_Δ_^rel^ values, which is in excellent agreement with the above theoretical analysis.

Comparing C2‐NH_2_ with C2‐NO_2_ reveals that C2‐NH_2_ has not only a higher *Φ*_f_ but also a higher ISC rate. This dual enhancement is mainly attributed to the modulation of TICT and PET. In Figure 2c, C2‐NO_2_ and C2‐NH_2_ exhibit the same pattern: the C2’‐aryl substituents, along with half of the Cy5 skeleton, serve as an electron‐withdrawing group or “acceptor” (gaining an electron), while the other half of the Cy5 skeleton functions as an electron‐donating group or “donor” (losing an electron) in the TICT state. Incorporating an ‐NO_2_ group into the C2’‐aryl group significantly strengthens the “acceptor,” resulting in a pronounced TICT tendency. In contrast, attaching an ‐NH_2_ group to the same position reduces the strength of the “acceptor,” weakening the TICT tendency, which substantially improves the quantum yield of C2‐NH_2_. Additionally, C2‐NH_2_ exhibits a modestly enhanced tendency for PET—a process highly efficient in promoting ISC. Consequently, C2‐NH_2_ exhibits a significantly higher ISC yield. However, it is important to mention that both PET and TICT, while enhancing ISC (to different degrees), also quench fluorescence, resulting in a lower *Φ*_f_ of C2‐NH_2_ (32%) and C2‐NO_2_ (4%) compared with that of the reference compound TCy5‐H (56%).

Subsequently, the PET and TICT tendencies of the C2’‐ and meso‐substituted Cy5 analogs were examined (Figures S12–S19, Supporting Information). When an electron‐donating group (–NH_2_) was attached to form meso‐NH_2_, the activation of both PET and TICT was observed like that in the case of C2‐NH_2_. However, one key difference between C2‐NH_2_ and meso‐NH_2_ is the relative stabilities of their ET and TICT states. For C2‐NH_2_, the ET state was more stable than the TICT state by 0.13 eV, and so, a greater number of C2‐NH_2_ molecules were expected to enter the ET state, which is highly efficient in populating the triplet states via ISC. By contrast, in meso‐NH_2_, the TICT state is more stable than the ET state by 0.06 eV. Since the TICT state is highly susceptible to internal conversion, a lower triplet formation yield was expected for meso‐NH_2_. Unfortunately, the synthesis of meso‐NH_2_ is highly challenging, and we were unable to produce this compound to substantiate our computational predictions.

Overall, the computational results indicated that C2’‐substituted Cy5 derivatives offer a highly versatile platform for effectively tuning the PET/TICT tendencies and generating efficient photosensitizers by introducing a strongly electron‐donating group to the aryl ring attached at the C2’ position.

### 2.4 Ultrafast Spectroscopy

**Figure 4:**
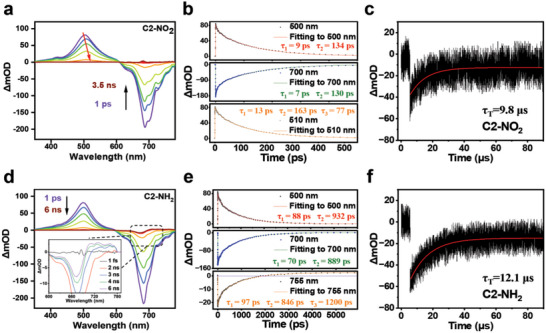
Experimental results of ultrafast spectroscopy and kinetic fitting results. a) Femtosecond transient absorption spectra of C2‐NO_2_ in DCM (λ_ex_ = 680 nm). b) Kinetic traces and fitting lines of C2‐NO_2_ at the representative ESA and GSB wavelengths. c) Decay trace recorded at 685 nm for C2‐NO_2_ excited at 610 nm (10 µM in deaerated DCM) at 25 °C. d) Femtosecond transient absorption spectra of C2‐NH_2_ in DCM (λ_ex_ = 680 nm). e) Kinetic traces and fitting lines of C2‐NH_2_ at representative ESA and GSB wavelengths. f) Decay trace recorded at 674 nm for C2‐NH_2_ excited at 610 nm (10 µM in deaerated DCM) at 25 °C.

To verify the excited state kinetics of the decay pathway, the femtosecond transient absorption spectroscopy spectra of the C2’‐position aryl‐modified Cy5 dyes were recorded in DCM. As shown in **Figure** **4**a, the excited state absorption bands (ESA) band feature of C2‐NO_2_ at 500 nm can be attributed to the S_1_‐S_n_ absorption, while the negative signal at 700 nm may be the result of ground state bleaching (GSB) of the S_1_‐S_0_ absorption. Over time, the maximum ESA peak became red‐shifted from 500 nm to 510 nm, likely due to the TICT process.^[^ ^19^ ^]^ Thus, wavelengths of 500, 700, and 510 nm were selected for kinetic fitting (Figure 4b), wherein τ = 9 and 7 ps were attributed to nonradiative processes, while τ = 134 and 130 ps were attributed to the emissive ICT state because of their comparable lifetimes. However, a new process appeared at 510 nm with a lifetime of 77 ps, which was due to the TICT state, as it is comparable to that of a fully charge‐separated excited state reported in a previous study.^[^ ^20^ ^]^

Subsequently, C2‐NH_2_ was subjected to a similar analysis, as presented in Figure 4d. This dye also produced ESA and GSB bands at 500 and 700 nm, but unlike C2‐NO_2_, the ESA of C2‐NH_2_ did not show a redshift, indicating that it did not undergo TICT. However, C2‐NH_2_ exhibited a new ESA band at 755 nm, so wavelengths 500, 700, and 755 nm were selected for kinetic fitting. As shown in Figure 4e, a new process (τ = 1200 ps) appeared at 510 nm. Since the triplet‐state ESA of Cy5 is usually located at 700–800 nm,^[^ ^21^ ^]^ the long lifetime process can be attributed to the ISC.

Subsequently, the T_1_ lifetimes of the C2’‐position aryl‐modified Cy5 dyes were investigated in degassed DCM using laser flash photolysis spectrophotometry. As shown in Figure 4c,f, C2‐NH_2_ exhibited a distinct triplet state signal that is stronger than those of C2‐NO_2_ and C2‐OMe (Figure S21, Supporting Information). This strongest triplet signal further confirms that C2‐NH_2_ has the highest ISC efficiency. Furthermore, the triplet lifetimes (τ_T_) of the dyes were obtained by fitting the triplet decay curves (Figure 4c,f, Table 1), and the triplet state lifetime of C2‐NH_2_ was measured to be 12.1 µs (Figure 4f). Such a long lifetime is sufficient to endow C2‐NH_2_ with an outstanding photosensitizer performance, enabling it to generate ROS through electron transfer or energy transfer processes with the O_2_ present in the surrounding microenvironment.

### 2.5 NTR‐Mediated Activation Test

**Figure 5:**
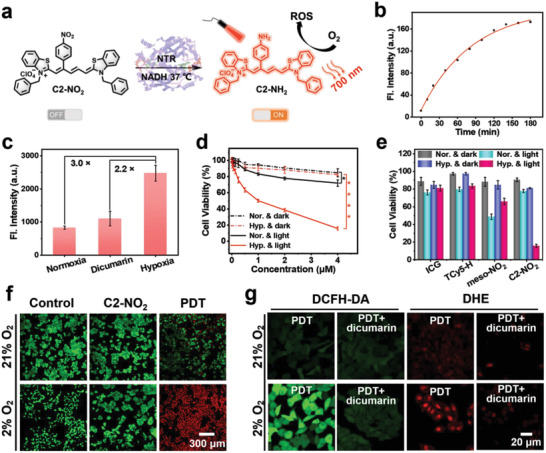
Verification of the evolution of C2‐NO_2_ to C2‐NH_2_ in aqueous solvent and cells. a) Schematic illustration of the conversion of C2‐NO_2_ to C2‐NH_2_ in the presence of NTR. b) Fluorescence intensity of C2‐NO_2_ (10 µM) treated with NTR (10 µg mL^−1^) as a function of time. c) Fluorescence intensities of the HepG2 cells incubated with C2‐NO_2_ (5 µM) under normoxic, hypoxic with dicumarin pretreatment, and hypoxic conditions (*n* = 3). d) Cytotoxicity of C2‐NO_2_ toward HepG2 cells in the dark and with light irradiation (660 nm, 20 mW cm^−2^, 15 min) under normoxic and hypoxic conditions. e) Cytotoxicities of different dyes (4 µM) toward HepG2 cells in the dark and with light irradiation (660 nm, 20 mW cm^−2^, 15 min) under normoxic and hypoxic conditions. f) Imaging of the calcium‐AM/PI staining of cells after different treatments. g) Intracellular ROS production by C2‐NO_2_ in HepG2 cells monitored by the DCFH‐DA and DHE assays under different conditions. Data are expressed as the mean ± standard deviation (SD) *p < 0.1, **p < 0.01, ***p < 0.001, and ****p < 0.0001, as determined by Student's t‐test.

NTR is a representative reductase present in most tumor cells. This enzyme could effectively reduce nitro groups to amino groups in the presence of nicotinamide adenine dinucleotide (NADH). The expression of NTR in the cell was related to O_2_ content, the hypoxia microenvironment can induce the overexpression of NTR, and the catalytic activity of nitro to amino can be enhanced.^[^ ^22^ ^]^ Interestingly, while C2‐NO_2_ has low *Φ*_f_ and *Φ*_Δ_^rel^ values, C2‐NH_2_ exhibits high efficiencies in generating both fluorescence and ROS (Table 1). Thus, C2‐NO_2_ was employed as a precursor to the photosensitizer, which can be activated in hypoxic tumor cells to produce C2‐NH_2_ by NTR and NADH. This activation could lead to strong fluorescence and significant amounts of ROS production for the image‐guided PDT of hypoxic tumors (**Figure** **5**a).

The sensitivity and specificity of C2‐NO_2_ to NTR were then investigated to verify the activatable properties of the hypoxic microenvironment. As expected, the addition of NTR and NADH successfully triggered the transition from C2‐NO_2_ to C2‐NH_2_, resulting in a significant increase in fluorescence at 690 nm (16‐fold, Figure 5b) after the enzyme‐catalyzed reactions (120 min, Figure S23a, Supporting Information). Notably, the fluorescence was enhanced 6.7‐fold upon increasing the concentration of NTR from 0 to 10 ug mL^−1^ (Figure S23b, Supporting Information). To further validate the mechanism of nitro to amino conversion, C2‐NO_2_, NTR, and NADH were incubated together in an aqueous solution at 37 °C for 2 h and the resulting mixture was analyzed using HPLC. The co‐incubation solution showed a peak with the same retention time as that of C2‐NH_2_ (Figure S24, Supporting Information), indicating the successful conversion of C2‐NO_2_ to C2‐NH_2_. To further assess the selectivity, the response of C2‐NO_2_ to the different metal ions, amino acids, and reducing substances in the cells was studied. Under the same conditions, fluorescence enhancement occurred only in the presence of NTR and NADH (Figure S25, Supporting Information), thereby demonstrating the outstanding specificity of the photosensitizer precursor.

Molecular docking was also performed at the atomic level to simulate possible interactions and binding mechanisms. The X‐ray crystal structure of NTR (1.50 Å resolution, protein data bank 4DN2) with one protein chain containing 381 residues was obtained from the Protein Data Bank. Through the analysis of results, it was found that the nitrobenzene moiety of C2‐NO_2_ was able to enter and bind tightly to the protein structural domain (Figure S26, Supporting Information, amino acid residues: ASN‐41, SER‐38, ARG‐72) and exhibited a great affinity to NTR, as reflected by the low thermodynamic binding energy of −6.95 kcal mol^−1^.

### 2.6 In Vitro Cellular Imaging and Cytotoxicity Under Hypoxia

Encouraged by the results obtained under aqueous conditions, C2‐NO_2_ was applied in several in vitro cellular experiments. As a cationic small molecule, C2‐NO_2_ rapidly penetrated and accumulated in the HepG2 cells within 1 h of incubation (Figure S27, Supporting Information). Subsequently, the intracellular distribution of C2‐NO_2_ was probed using commercial organelle‐selective trackers. It was found that the red fluorescence of C2‐NO_2_ overlapped well with the green fluorescence of the mitochondria label Mito Tracker Green (Pearson's coefficient: 0.91), whereas poor overlap was observed with the blue fluorescence of the Hoechst 33342 nucleus tracker (Pearson's coefficient: −0.09) and the green fluorescence of the Lyso‐Tracker Green lysosome tracker (Pearson's coefficient: 0.23) (Figure S28, Supporting Information). These results show that C2‐NO_2_ is predominantly enriched in the mitochondria, most likely due to electrostatic interactions between the positive charge of C2‐NO_2_ and the negative potential of the outer mitochondrial membrane.

Moreover, the activation of C2‐NO_2_ in the cytosol was verified. C2‐NO_2_ was incubated with HepG2 cells in normoxia (21% O_2_), hypoxia (2% O_2_), and hypoxic cells pre‐cultured with dicumarin (an inhibitor known as NTR)^[^ ^23^ ^]^ for 1 h. The imaging results showed that the bright red fluorescent occurred in the hypoxic cells, which is 4.9 times and 2.5 times higher than that in normoxic and dicumarin‐pretreated hypoxic cells (Figure 5c and Figure S29, Supporting Information), thereby confirming the importance of hypoxia in the nitro‐to‐amino conversion.

Subsequently, the in vitro eradication of cancer cells by C2‐NO_2_ under normoxic and hypoxic conditions was examined using the methyl thiazolium tetrazolium (MTT) assay. As shown in Figure 5d, the elimination of HepG2 cells under normoxic conditions was difficult in both the dark and light groups (660 nm, 20 mW cm^−2^, 15 min). By contrast, under hypoxic conditions, the light group effectively induced HepG2 cell disruption, reducing the cell viability to <20%. Furthermore, ICG, TCy5‐H, and meso‐NO_2_ were tested as controls and were found to exhibit poor phototoxicities under both normoxic and hypoxic conditions. For example, in the meso‐NO_2_ group, the cell viability remained >50% under normoxic conditions and ≈60% under hypoxic conditions (Figure 5e; Figures S30 and S31, Supporting Information). Furthermore, 4T1, A549, and MCF7 cells were employed to carry out the MTT assay. In these cases, a low dark toxicity (cell viability > 80%) and a high phototoxicity (cell viability <25%) were observed after the co‐incubation of C2‐NO_2_ with different cell lines in a hypoxic environment (Figure S32, Supporting Information). However, in normal cells (3T3 and L‐O2) of normoxic, C2‐NO_2_ exhibited low phototoxicity (cell Viability > 70%) and low dark toxicity (cell Viability > 80%), demonstrating that phototoxicity of C2‐NO_2_ was only turned on in hypoxic tumor cells. (Figure S33, Supporting Information). Calcein acetoxymethyl ester/propidium iodide (Calcein‐AM/PI) cell fluorescence (live/dead) staining yielded comparable results. More specifically, in the control group, only green fluorescence was observed in the live HepG2 cells under both normoxic and hypoxic conditions. For the PDT group of normoxic cells, the mixture of green and red fluorescence was detected, indicating the weak ability of C2‐NO_2_ to damage cells under 660 nm light irradiation (20 mW cm^−2^, 15 min). As expected, in the PDT group of hypoxic cells, only red fluorescence was observed because C2‐NO_2_ was converted to C2‐NH_2_ under hypoxic conditions, effectively killing the HepG2 cells (Figure 5f).

The 660 nm LED‐induced intracellular generation of ROS was then explored under normoxic and hypoxic conditions using 2,7‐dichlorodihydrofluorescein diacetate (DCFH‐DA) mediated fluorescence (Figure 5g). Following PDT (660 nm, 10 mW cm^−2^, 5 min), only weak green fluorescence was observed in the normoxic cells, whereas bright green fluorescence was observed in the hypoxic cells. By contrast, after pretreatment with dicumarin, weak fluorescence was observed in both the normoxic and hypoxic cells. In addition, the generation of O_2_^•−^ was detected by DHE staining, and bright red fluorescence was observed exclusively in the hypoxic cells (Figure 5g). These results indicated that in the hypoxic environment, the excellent ISC efficiency of C2‐NH_2_ still ensured the generation of large amounts of ROS to eradicate tumor cells. NO and ONOO^−^which may affect cell activity were shown not to be present in the system (Figure S34, Supporting Information). Moreover, JC‐1 staining of the mitochondrial membrane potential showed that severe mitochondrial depolarization occurred in the C2‐NO_2_ group upon 660 nm irradiation under hypoxic conditions (Figure S35, Supporting Information).

### 2.7 Therapeutic Effects In Vivo

**Figure 6:**
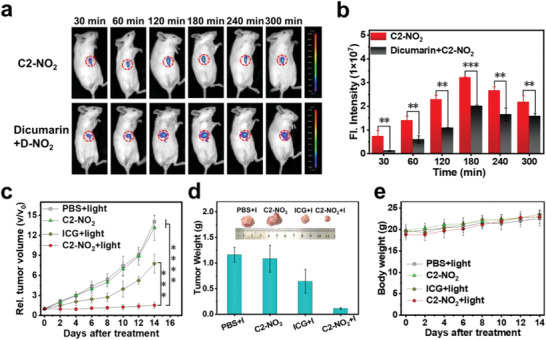
In vivo subcutaneously solid tumor PDT evolution of C2‐NO_2_. a) In vivo fluorescence imaging of tumor‐bearing mice after different treatments. b) fluorescence intensities of the tumors in mice injected with C2‐NO_2_ and dicumarin plus C2‐NO_2_ over time. c) Relative tumor volume of mice over time upon different treatments (*n* = 5). d) Tumor weights and photographic images of tumors of varying sizes following treatments (*n* = 5). e) Body weights of the mice over time after different treatments (*n* = 5). Data were expressed as mean ± SD *p < 0.1, **p < 0.01, ***p < 0.001, and ****p < 0.0001 determined by Student's t‐test.

Considering these promising in vitro results, the feasibility of using C2‐NO_2_ for tumor treatment in vivo was evaluated. For this purpose, a solid tumor model was established by injecting 4T1 cells into the axillae of BALB/c mice. Tumor fluorescence imaging was performed at various time points once the tumor diameter reached ≈100 mm^3^. As shown in **Figure** **6a,b**, direct C2‐NO_2_ injection led to significantly stronger NIR fluorescence at all time points compared to that observed in the dicumarin‐treated group; however, both groups reached a maximum at ≈3 h post‐injection. These findings demonstrate that after the in‐situ injection of C2‐NO_2_ into the tumor site, the hypoxic tumor microenvironment promotes the reduction of nitro to amino groups by NTR and NADH.

The anti‐tumor properties of C2‐NO_2_ were then tested in 4T1 tumor‐bearing mice. The mice were randomly divided into four groups (*n* = 5), namely the PBS+light, C2‐NO_2_ alone, ICG+light, and C2‐NO_2_+light groups. The therapeutic effect of each treatment on the tumors was assessed by monitoring the relative volume of each axillary tumor in the mice models. As shown in Figure 6c,d, the volumes of the tumors treated with PBS+light and C2‐NO_2_ alone clearly increased (i.e., by 14.1‐fold and 13.1‐fold, respectively). By contrast, the tumors in the ICG+light treatment groups were moderately inhibited after 671 nm irradiation (100 mW cm^−2^, 15 min), although the tumor volume still increased to 7.8‐fold after 14 d of treatment. Remarkably, tumor growth following the combination of C2‐NO_2_ treatment with 671 nm irradiation (100 mW cm^−2^, 15 min) was represented by only a 1.5‐fold growth after 14 d, thereby demonstrating that this treatment effectively inhibited tumor growth. Importantly, no significant weight loss was observed in any of the four groups of mice during the treatment period. Subsequently, all four groups of mice were sacrificed, and hematoxylin and eosin (H&E) staining was performed for histological analysis (Figure S36, Supporting Information). Pronounced cell destruction was observed in the tumors treated with C2‐NO_2_ and light, and no necrosis or inflammatory lesions were observed in the major organs (i.e., heart, liver, spleen, lungs, and kidneys). All mice showed very few abnormal changes in body weight during treatment (Figure 6e; Figure S37, Supporting Information), indicating that C2‐NO_2_ is both biocompatible and suitable for use in in vivo systems.

## 3 Conclusion

In summary, a pentamethyl cyanine platform was developed using the C2’‐aryl substitution strategy. By altering the electron‐donating/withdrawing strength of the substituents attached at the para‐position of the C2’‐aryl group, it was possible to effectively modulate the occurrence of both twisted intramolecular charge transfer (TICT) and photoinduced electron transfer (PET), thereby regulating the fluorescence intensities and intersystem crossing (ISC) efficiencies. More specifically, it was found that electron‐withdrawing substituents enhance TICT, resulting in efficient fluorescence quenching. As the electron‐donating ability of the substituent is increased, the TICT tendency was found to weaken significantly, while PET was enhanced. This change improved both the fluorescence quantum yield and the intersystem crossing (ISC) efficiency. To demonstrate the utility of this biocompatible cyanine platform, we employed C2‐NO_2_ as an imaging and photosensitizer precursor. As a result, it was found that the hypoxic microenvironment present in tumor cells can convert C2‐NO_2_ to C2‐NH_2_ in the presence of nitroreductase, thereby leading to bright fluorescence and the generation of large amounts of reactive oxygen species (ROS) for effective photodynamic therapy. We anticipate this C2’‐aryl‐substituted cyanine platform will enable sophisticated strategies to modulate diverse photophysical mechanisms and meet the demanding performance criteria of various image‐guided therapies.

## Conflict of Interest

The authors declare no conflict of interest.

## Supporting information

Supporting Information

## Data Availability

The data that support the findings of this study are available from the corresponding author upon reasonable request.
