# Supplementary material for: Aryl‐Modified Pentamethyl Cyanine Dyes at the C2’ Position: A Tunable Platform for Activatable Photosensitizers
Source: Adv Sci (Weinh). 2023 Dec 8;11(7):2305761. doi: 10.1002/advs.202305761 (PMC10870032; doi:10.1002/advs.202305761)
Supplement: Supplementary file 1 — Supporting Information [file ADVS-11-2305761-s001.pdf]

ADVANCED SCIENCE
Open Access

Supporting Information

for *Adv. Sci.*, DOI 10.1002/advs.202305761

Aryl-Modified Pentamethyl Cyanine Dyes at the C2’ Position: A Tunable Platform for Activatable Photosensitizers

Fuping Han, Syed Ali Abbas Abedi, Shan He, Han Zhang, Saran Long, Xiao Zhou, Supphachok Chanmungkalakul, He Ma, Wen Sun, Xiaogang Liu, Jianjun Du*, Jiangli Fan and Xiaojun Peng*

Supporting Information

Aryl-Modified Pentamethyl Cyanine Dyes at the C2′ Position: A Tunable Platform for Activatable Photosensitizers

Fuping Han[a], Syed Ali Abbas Abedi[b], Shan He[c], Han Zhang[a], Saran Long[a], Xiao Zhou[a], Sphachok Chanmungkalakul[b], He Ma[a], Wen Sun[a,d], Xiaogang Liu*[b], Jianjun Du*[a,d], Jiangli Fan[a,d], and Xiaojun Peng[a],

[a] State Key Laboratory of Fine Chemicals, Dalian University of Technology, Dalian 116024, China
[b] Fluorescence Research Group, Singapore University of Technology and Design, 487372, Singapore
[c] Department of Chemistry, Hong Kong Branch of Chinese National Engineering Research Center for Tissue Restoration and Reconstruction and Institute for Advanced Study, The Hong Kong University of Science and Technology, Clear Water Bay, Kowloon, Hong Kong, China
[d] Ningbo Institute of Dalian University of Technology, Dalian University of Technology, Ningbo 315016, China

*e-mail: dujj@dlut.edu.cn; Xiaogang_liu@sutd.edu.sg

1. General Information

In this report, the general chemicals used in the report were purchased from Energy Chemical Co., Bide Pharmatech Ltd., and J&K Scientific Ltd., and all the solvents were of analytic grade. NTR (nitroreductase) and NADH (Nicotinamide adenine dinucleotide) were purchased from Sigma-Aldrich. DCFH-DA (2, 7-dichlorofluorescein diacetate) Detection Kit and JC-1 Detection Kit were purchased from Beyotime Biotechnology Co. (China). All the other solvents and reagents used in

this study were of analytical grade.

NMR spectra were detected by Bruker Avance III 500 spectrometer or Bruker Avance III 400 spectrometer. Chemical shift (δ) was reported as ppm in DMSO-d6 with TMS as the internal standard. The mass spectrum (ESI-MS) date was recorded on LTQ Orbitrap XL instruments. Absorption and emission spectra for all the compounds were performed with a Lambda 35 UV-visible spectrophotometer (PerkinElmer) and a VAEIAN CARY Eclipse fluorescence spectrophotometer (Serial No. FL0812-M018), respectively. Fluorescence quantum yield was obtained with the HAMAMATSU absolute fluorescence quantum yield spectrometer (Serial No. C11347). Nanosecond time-resolved transient absorption spectra were recorded on a LP980 laser flash photolysis spectrometer (Edinburgh Instruments Ltd.) in combination with a Nd:YAG laser (Surelite I-10, Continuum Electro-Optics, Inc.). Confocal laser scanning microscope (CLSM) images were performed on Olympus FV3000 confocal laser scanning microscope. Small animals' fluorescence imaging was carried out by NightOWL II LB983 living imaging system.

2. Analysis Test Procedure

Photostability Detection

The absorbance of Cy5 compounds at the 660 nm was dissolved in water/DMSO (7:3). The cuvette was irradiated with 660 nm monochromatic light for various time, and absorption spectra were measured immediately.

Singlet Oxygen Detection

We used 1,3-diphenylisobenzofuran (DPBF) to measure singlet oxygen produced by cyanine dyes. The absorbance of DPBF at 415 nm was adjusted to about 1.0 and the absorbance of the dye to be tested at 660 nm was adjusted to about 0.35-0.55 in dichloromethane (3 mL). The cuvette was irradiated with 660 nm (5 mW cm^{-2}) monochromatic light for various time, and absorption spectra were measured immediately. Φ_{Δ}^{rel} was calculated by the following equation:

$$\boldsymbol{\Phi}_{\Delta sam} = \boldsymbol{\Phi}_{\Delta std} \left(\frac{k_{sam}}{k_{std}}\right) \left(\frac{F_{std}}{F_{sam}}\right)$$

Where "sam" and "std" represent the "PSs" and "ICG", respectively. "k" is the slope of absorbance change curve of DPBF at 415 nm, $F=1-10^{-O.D.}$ (O.D. is the absorbance of the solution at 660 nm).

At the same time, we used Singlet Oxygen Sensor Green (SOSG) to detect $^1O_2$ production in aqueous solution. Cyanine dyes and SOSG were prepared as 5 μM and 1 μM in water, respectively. The cuvette was irradiated by 660nm (5 mW cm^{-2}) monochromatic light for various time, and the fluorescence spectra were measured immediately. As control, SOSG aqueous solution without photosensitizers was subjected to irradiation (λ_{ex} = 488 nm).

Superoxide Anion Radical ($O_2^{\bullet-}$) Detection

We used dihydrorhodamine 123 (DHR123) to detect $O_2^{\bullet-}$ production, DHR123 can be converted to Rhodamine 123 in the presence of $O_2^{\bullet-}$. Cyanine dyes and DHR123 were prepared as 5 μM and 10 μM in water, respectively. The cuvette was

irradiated by 660nm (5 mW cm^{-2}) monochromatic light for various time, and the fluorescence spectra were measured immediately. As control, DHR123 aqueous solution without photosensitizers was subjected to irradiation (λ_{ex} = 488 nm).

At the same time, we also used Droethidium (DHE) as indicator for detection of $O_2^{\bullet-}$ in solution, When $O_2^{\bullet-}$ is generated in the system, DHE can be oxidized to form ethidium which intercalates into DNA and emits bright fluorescence at ~580 nm. 5 µM of cyanine dyes were dissolved in 3 mL water containing 15 µM of DHE and 100 µg/mL ctDNA. The mixture was then placed in a cuvette and irradiated with 660nm (10 mW cm^{-2}) monochromatic light for various time. The fluorescence change of sample was recorded by the fluorescence spectrometer.

Computational Methods

Density functional theory (DFT) and time-dependent DFT (TD-DFT) calculations were employed to elucidate the mechanism of cyanine compounds. The calculations were performed using Gaussian 16A software.[1] Geometry optimizations in both the ground and excited singlet states were conducted utilizing the ωB97XD functional with the def2-SVP basis set in dichloromethane.[2] To validate the stability of the structures on the potential energy surfaces (PES), frequency analysis was carried out. The solvent effects were considered using the SMD model,[3] and the electronic energies were calculated using the corrected linear response (cLR) solvent formalism.

For the modeling of the twisted intramolecular charge transfer (TICT) mechanism,[4] two dihedral angles along the rotation bond were systematically varied from approximately 0 (or 180; planar) to 90 degrees (perpendicular), with a step size of about 10 degrees. The remaining parameters were freely optimized, and the energies of these geometries were further corrected using the corrected linear

solvation formalism. To account for potential state-crossing between the S_1 and S_2 states, optimization of both states was performed to construct the S_1 potential energy surface (PES). From this PES, the energy barriers of rotation and the driving energy required to enter the TICT state were extracted.

During the modeling of the photon-induced electron transfer (PET) mechanism,[5] the phenyl ring at the C2'-position was fixed at a 90-degree angle to ensure complete charge transfer (or electron transfer). Subsequently, the electronic energy was corrected using the corrected linear solvation formalism.

In most cases, the triplet state energy was calculated using the DFT method based on the corresponding optimized singlet states. In situations involving multiple triplet states, such as both T_1 and T_2, TD-DFT calculations were performed on the geometries of the optimized singlet excited state, along with the corrected linear solvation formalism, to determine the energy levels of the triplet state.

Spin-orbit coupling values were calculated using ORCA 5.0 software,[6] employing the ωB97XD functional with the def2-SVP basis set in dichloromethane. The SMD model was utilized to account for solvent effects.

Femtosecond transient absorption experiments

Femtosecond TA experiments. Femtosecond TA experiments were based on a Pharos laser (1030 nm, 100 kHz, 230 fs pulse-duration; Light Conversion) and Orpheus-HP optical parameter amplifier (OPA; Light Conversion). The 1030-nm output laser was split into two beams with 80/20 ratio. The 80% parts was used to pump the OPA to generate a wavelength tunable pump beam. The 20% parts was further split into two parts with 75/25 ratio. The 75% parts was attenuated with a neutral density filter and focused into a BBO crystal to generate a 515 nm beam, which was further focused into a Sapphire crystal to generate a white light continuum used as the probe beam. The probe beam was focused with an Al parabolic reflector onto the sample. After the sample, the probe beam was collimated and then focused

into a fiber-coupled spectrometer with CMOS cameras and detected at a frequency of 10 kHz. The pump pulses were chopped by a synchronized chopper at 5 kHz and the absorbance change was calculated with twenty adjacent probe pulses (ten pump-blocked and ten pump-unblocked). The intensity of the pump pulse used in the experiment was controlled by a variable neutral-density filter wheel. The delay between the pump and probe pulses was controlled by a motorized delay stage. The linear polarization angle difference between the pump and probe light keeps magic angle (54.7°) to record the isotropic response.

Nanosecond Time-resolved Transient Absorption Spectra

The triplet lifetimes of dyes were recorded on a LP980 laser flash photolysis spectrometer (Edinburgh Instruments Ltd.) in combination with a Nd:YAG laser (Surelite I-10, Continuum Electro-Optics, Inc.). Samples (10 μM) in deaerated DCM were excited by a 610 nm laser pulse (1 Hz, 100 mJ per pulse, fwhm ≈ 7 ns) at room temperature. The triplet state decay kinetics was measured at λ_{abs}.

Response of C2-NO_2 Toward NTR

Measurements of fluorescence spectra were performed in aqueous solution (PBS/DMSO = 8:2, v/v, 0.01 M, pH = 7.4). For fluorescence response experiments with different incubation times, in a 3.0 mL tube, aqueous solution, C2-NO_2 (10 μM), NADH (500 μM), and NTR (10 ug/mL), respectively. And adjust the final solution volume to 3 mL. Fluorescence spectra were recorded at different times. For fluorescence response experiments at different concentrations of NTR, in a 3.0 mL tube, aqueous solution, C2-NO_2 (10 μM), NADH (500 μM), and NTR (0-10 ug/mL), respectively. And adjust the final solution volume to 3 mL. The fluorescence spectra

were recorded after incubation for 2 h.

Cell and Culture Conditions

HepG2 cells, MCF7 cells and 4T1 cells were purchased from the Institute of Basic Medical Science (IBMS) of the Chinese Academy of Medical Sciences and cultured with Dulbecco's modified Eagle's medium (DMEM, Invitrogen), all of them were supplemented with 1% penicillin streptomycin and 10% fetal bovine serum, and atmosphere of CO_2/air = 5%/95% at 37 ºC.

Confocal Fluorescence Imaging of Cells

HepG2 cells were cultured for 24 hours. For cell colocalization assay, first add LysoTracker Green DND 26 (100 nM), MitoTracker Green FM (100 nM) or Hoechst 33342 (100 nM) to the culture medium and incubate for 30 min, then add C2-NO_2 (0.5 μM) and then observe the cells with confocal laser microscope. The excitation wavelength for C2-NO_2 is 660 nm, while the excitation wavelength for LysoTracker Green DND 26 and MitoTracker Green FM is 488 nm, and the excitation wavelength for Hoechst 33342 is 405 nm. The emission wavelength was collected from 690 to 740 nm for C2-NO_2, 500 to 540 nm for Lyso Tracker Green and Mito Tracker Green, and 440 to 480 nm for Hoechst 33342.

***In Vitro* Photo-Cytotoxicity Assays**

To simulate a hypoxic tumor environment, cells were cultured in an incubator at 37 °C in an atmosphere of 2% O_2, and the oxygen content in the chamber was

monitored using an oxygen detector (Nuvair, O Qucikstick).

HepG2 cells, MCF7 cells or 4T1 cells were seeded into 96-well plates at 5000 cells per well and incubated at 37 ºC for 24 hours. Cells were cultured for an additional 6 h under normoxia or hypoxia. For normoxic photocytotoxicity assessment, DMEM medium containing various concentrations of photosensitizers from 0 to 4 μM was added to wells culturing normoxic cells. For hypoxic photocytotoxicity assessment, DMEM medium containing different concentrations of photosensitizers 0 to 4 μM was added to the wells of hypoxic cells, respectively. Then, the cells were further cultured under normoxia or hypoxia for 2 hours, respectively. Subsequently, cells were irradiated with 660 nm light (20 mW cm^{-2} for 15 min). The cells were then incubated for a further 12 hours at 37 ºC. Next, add the formulated MTT-containing DMEM solution (5 mg/mL) to each well. After 4 h of cell culture, carefully remove the solution from each well, add 100 μL of DMSO to each well, and measure the absorbance at 540 nm and 620 nm with a Bio-Rad microplate reader, respectively. The viability was expressed as a percent of the controlled one using the following equation:

$$\textbf{Cell viability (\%)} = \frac{OD_{dye540}-OD_{dye620}}{OD_{control540}-OD_{concrol620}}\times 100\ \%$$

For dark toxicity measurement of different dyes, light irradiation step was canceled and all other steps were the same.

Live and Dead Cell Staining

HepG2 cells were cultured on cell culture plates for 24 h. Cells were cultured

under normoxic or hypoxic conditions for an additional 10 hours and then exposed to different following treatments: 1) normoxic cells; 2) normoxic cells were irradiated with 660 nm light (20 mW cm^{-2}, 15 min); 3) normoxic cells were incubated with 0.5 μM C2-NO_2 for 1 h and irradiated with 660 nm light (20 mW cm^{-2}, 15 min); 4) hypoxic cells; 5) hypoxic cells were irradiated with 660 nm light (20 mW cm^{-2}, 15 min); 6) hypoxic cells were incubated with 0.5 μM C2-NO_2 for 1 h and irradiated with 660 nm light (20 mW cm^{-2}, 15 min); After different treatments, calcein AM and PI co-staining was performed. The excitation wavelength is 488 nm, the emission wavelength is 505 to 545 nm for the green channel, and 600 to 700 nm for the red channel.

Intracellular ROS Detection

DCFH-DA (2,7-dichlorofluorescein diacetate) Detection Kit was used to verify ROS production in live HepG2 cells. Cells were first incubated on cell culture dishes for 24 h and then incubated under normoxia or hypoxia for 6 h. HepG2 cells were divided into 6 groups : 1) normoxic cells irradiated with 660 nm light (10 mW cm^{-2}, 5 min); 2) normoxic cells were incubated with 0.5 μM C2-NO_2 for 2 h and irradiated with 660 nm light (10 mW cm^{-2}, 5 min); 3) normoxic cells were incubated with 0.5 μM C2-NO_2 and 0.1 mM dicumarin for 2 h and irradiated with 660 nm light (10 mW cm^{-2}, 5 min); 4) hypoxic cells irradiated with 660 nm light (10 mW cm^{-2}, 5 min); 5) hypoxic cells were incubated with 0.5 μM C2-NO_2 for 2 h and irradiated with 660 nm light (10 mW cm^{-2}, 5 min); 6) hypoxic cells were incubated with 0.5 μM C2-NO_2 and

0.1 mM dicumarin for 2 h and irradiated with 660 nm light (10 mW cm^{-2}, 5 min). Use the DCFH-DA Detection Kit according to the manufacturer's instructions. Then, confocal luminescence imaging (excitation at 488 nm and monitoring at 490 to 520 nm) was performed.

DHE was used to verify $O_2^{\bullet-}$ production in live HepG2 cells. Cells were first incubated on cell culture dishes for 24 h and then incubated under normoxia or hypoxia for 6 h. HepG2 cells were divided into 6 groups : 1) normoxic cells irradiated with 660 nm light (10 mW cm^{-2}, 5 min); 2) normoxic cells were incubated with 0.5 μM C2-NO_2 for 2 h and irradiated with 660 nm light (10 mW cm^{-2}, 5 min); 3) normoxic cells were incubated with 0.5 μM C2-NO_2 and 0.1 mM dicumarin for 2 h and irradiated with 660 nm light (10 mW cm^{-2}, 5 min); 4) hypoxic cells irradiated with 660 nm light (10 mW cm^{-2}, 5 min); 5) hypoxic cells were incubated with 0.5 μM C2-NO_2 for 2 h and irradiated with 660 nm light (10 mW cm^{-2}, 5 min); 6) hypoxic cells were incubated with 0.5 μM C2-NO_2 and 0.1 mM dicumarin for 2 h and irradiated with 660 nm light (10 mW cm^{-2}, 5 min). Use the DHE according to the manufacturer's instructions. Then, confocal luminescence imaging (excitation at 488 nm and monitoring at 570 to 630 nm) was performed.

Mitochondria Disruption Test

HepG2 Cells were first incubated on cell culture dishes for 24 h and then incubated under normoxia or hypoxia for 6 h. HepG2 cells were divided into 6 groups : 1) normoxic cells were incubated with 0.5 μM C2-NO_2 for 1 h; 2) normoxic

cells were incubated with 0.5 μM C2-NO_2 for 1 h and irradiated with 660 nm light (10 mW cm^{-2}, 10 min); 3) hypoxic cells were incubated with 0.5 μM C2-NO_2 for 1 h; 4) hypoxic cells were incubated with 0.5 μM C2-NO_2 for 1 h and irradiated with 660 nm light (10 mW cm^{-2}, 10 min). Healthy cells with high mitochondrial membrane potential appear red (JC-1 is J-aggregates), while apoptotic cells with low mitochondrial membrane potential appear green (JC-1 is J-aggregates).

Subcutaneous Tumor Model and *In Vivo* Imaging

All animal operations were in accordance with institutional animal use and care9 regulations approved by the Model Animal Research Center of Dalian Medical University (MARC). Female BALB/c mice, 5 weeks old, were purchased from Liaoning Changsheng Biotechnology co. Ltd. mouse solid tumor model was then established by subcutaneous injection of 5×10^6 4T1 cells at selected axillary locations. Tumors were allowed to grow to a volume of approximately 100 mm^3. The mouse model construction and imaging experiments involved in this work were carried out under Guide for the Care and Use of Laboratory Animals (8th edition) published by the US National Institutes of Health in 2011, all manipulations were followed by USA National Research Council regulation. The animal protocol was approved by the local research ethics review board of the Animal Ethics Committee of Dalian University of Technology (2019-016).

For *in vivo* tumor imaging, 4T1 tumor-bearing BALB/c mice were divided two groups: 1) injecting C2-NO_2 (50 μmol, 100 μL) into the tumor; 2) preinjecting

dicumarin (100 μmol, 100 μL) into the tumor for 1h and then $C2\text{-}NO_2$ (50 μmol, 100 μL) was injected. The fluorescence signals were monitored at different post injection time (30 min, 60 min, 120 min, 180 min, 240 min and 300 min).

In Vivo Solid Tumor Phototherapy Evaluation

To evaluate the effect of photodynamic treatment with $C2\text{-}NO_2$ in vivo, mice were divided into four groups of five tumor-bearing mice each: 1) saline injection and irradiation; 2) ICG (50 μmol, 100 μL) injection and irradiation; 3) $C2\text{-}NO_2$ injection; and 4) $C2\text{-}NO_2$ (50 μmol, 100 μL) injection and irradiation. After 3 h of injection, the tumor areas were irradiated with 671 nm laser (100 mW cm^{-2}, 15 min). After different treatments, the volume (V) of the tumor was calculated according to the following formula: $V = a\times b^2 / 2$, where a represented the maximum diameter (mm) among solid tumor diameters, and b represented the diameter (mm) perpendicular to a. The tumor growth curve was calculated by comparing the tumor volume (V) with the initial tumor volume (V_0). Mice were executed after 14 d of treatment, and tumor tissues were removed and stained with hematoxylin-eosin (H&E) for histological analysis.

In Vivo Biosafety Assay

The *in vivo* biosafety assay was performed by measuring the body weight of mices and histological analysis of H&E sections. Mice were executed after 14 d of treatment, and the major organs, such as heart, liver, spleen, lung, and kidney, were removed and stained with hematoxylin-eosin (H&E) for histological analysis.

3. Synthesis of cyanine dyes

According to the literature methods. **TCy5-H**, **meso-CF_3**, **meso-OMe** and **meso-NO_2** were prepared. [7]

1)

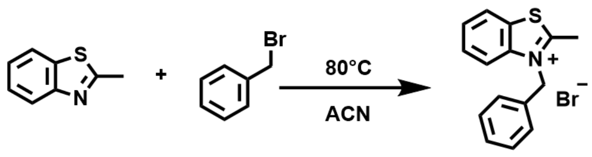

1

Scheme S1. Preparation of compounds **1**

Synthesis of **1:**

Benzyl bromide (4.6 g, 26.8 mmol) was added to a stirred solution of 2-methylbenzothiazole (2.0 g, 13.4 mmol) in dry acetonitrile (10 mL). The reaction was heated to 60 °C under N_2 atmosphere and stirred for 24 h. Subsequently, the reaction was allowed to cool to room temperature. The precipitate was filtered and washed with ethyl acetate and dried under vacuum to afford compound 1 as a light green solid in 88.5% yield (3.8 g, 11.9 mmol).

2)

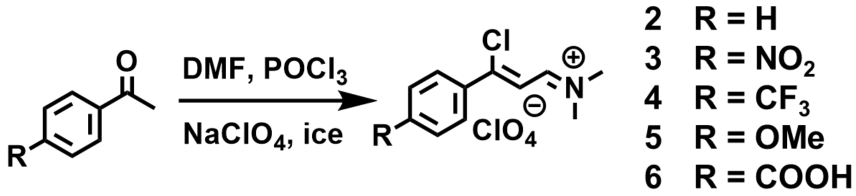

Scheme S2. Preparation of compounds **2-6**

Synthesis of **2**

$POCl_3$ (3 mL, 32.1 mmol) was added dropwise to dry DMF (15 mL) at 0 °C. The

mixture was stirred at room temperature for 3 h. Subsequently, acetophenone (1 g, 8.32 mmol) was added to the reaction solution. The red solution formed was heated at 75°C for 12 h. After cooling to room temperature, the solution was poured into 100 mL of ice water and then 50 ml of saturated $NaClO_4$ solution was added. The solid obtained was filtered and washed twice with cold aqueous water and finally dried under vacuum. The product **2** was obtained as a yellow solid (1.3 g, 51.6% yield). HRMS(ESI): m/z calc. for $[C_{11}H_{13}ClN]^+$ 194.0731, found 194.0732 $[M\text{-}ClO_4]^+$. **2** can be used in the next reaction without further purification.

Synthesis of **3-6**

Compound **3-5** were synthesized according to the synthetic procedure of compound **2**. The compound **3** was gotten as brown white solid (0.7 g, 33.2 % yield). HRMS(ESI): m/z calc. for $[C_{11}H_{12}ClN_2O_2]^+$ 239.0582, found 239.0584 $[M\text{-}ClO_4]^+$. The compound **4** was gotten as white solid (0.9 g, 45.7 % yield). HRMS(ESI): m/z calc. for $[C_{12}H_{12}ClF_3N]^+$ 262.0605, found 262.0607 $[M\text{-}ClO_4]^+$. The compound **5** was gotten as saffron yellow solid (1.3 g, 61.2 % yield). HRMS(ESI): m/z calc. for $[C_{12}H_{13}ClNO_2]^+$ 238.0629, found 238.0625 $[M\text{-}ClO_4]^+$. The compound **6** was gotten as saffron yellow solid (1.4 g, 63.2 % yield). HRMS(ESI): m/z calc. for $[C_{12}H_{15}ClNO]^+$ 224.0837, found 224.0836 $[M\text{-}ClO_4]^+$.

Synthesis of 7

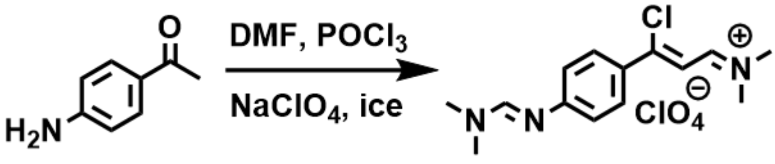

Scheme S3. Preparation of compounds **7**

he synthesis of **compound 7** follows the same procedure as that of **compounds 3-6.** However, due to the reaction system being acidic, the amino group reacts with DMF to form a Schiff base. As a result, the para-substituent in **compound 7** is not an amino group, but rather a Schiff base. The **compound 7** was gotten as yellow solid (1.4 g, 63.2 % yield). HRMS(ESI): m/z calc. for $[C_{14}H_{19}ClN_3]^+$ 264.1262, found 264.1265 $[M\text{-}ClO_4]^+$.

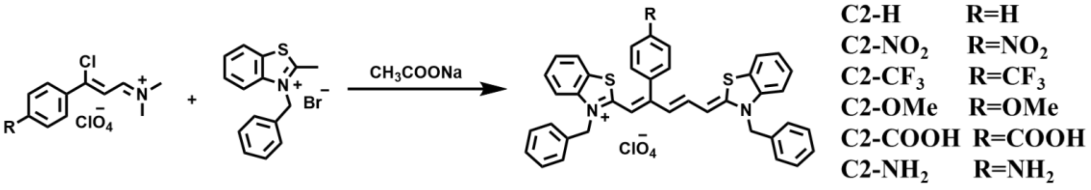

Scheme S4. Preparation of the C2'-modification cyanine dyes

Synthesis of **C2-H**

Compound **3** (0.1 g, 0.3 mmol) and compound **1** (0.25 g, 0.76 mmol) were added to acetic anhydride (10 ml) and stirred at 40°C until the solid was completely dissolved. Then 0.1 g of CH_3COONa was added to catalyze the reaction and the solution was reacted at 80°C and monitored by TLC analysis. After a significant reduction of the yellow intermediate, the solvent was evaporated under reduced pressure to produce a residue. The crude material was adsorbed on silica gel and purified by column chromatography (dichloromethane/methanol= 100/1), obtained a blue solid **D-H** (130 mg, 58.6 % yield). The solid was redissolved in DMSO / diethyl ether and stored in 25°C. Blue crystals were obtained suitable for X-ray diffraction analysis after two weeks. CCDC 2246197 contain the crystallographic data for this paper. These data can be obtained free of charge from The Cambridge

Crystallographic Data Centre. **^{1}H NMR** (500 MHz, DMSO-*d6*): δ = 7.89 (d, 1H), 7.83 (d, 1H), 7.73 (d, 1H), 7.68 (t, 2H), 7.61 (d, 1H), 7.50 (t, 1H), 7.45 (t, 1H), 7.40 (t, 2H), 7.36 (d, 2H), 7.32 (d, 2H), 7.27 (m, 4H), 7.23 (d, 2H), 6.84 (s, 1H), 6.70 (s, 1H), 6.68 (s, 1H), 6.58 (s, 1H), 5.80 (s, 2H), 5.59 (s, 2H) ppm; **^{13}C NMR** (100 MHz, DMSO-*d6*): δ = 140.60, 135.21, 130.75, 130.44, 129.96, 129.55, 129.46, 129.38, 128.75, 128.49, 128.42, 127.10, 127.07, 125.83, 125.39, 125.26, 123.66, 123.21, 114.07, 113.64, 55.38, 49.80, 49.07 ppm; HRMS(ESI): m/z calc. for $[C_{39}H_{31}N_2S_2]^+$ 591.1923, found 591.1925 $[M\text{-}ClO_4]^+$.

Synthesis of **C2-NO$_2$**

C2-NO$_2$ was synthesized according to the synthetic procedure of **C2-H**. The product obtained was a green powder (91 mg, 44.2 % yield). **^{1}H NMR** (500 MHz, DMSO-*d6*): δ = 8.52 (d, 2H), 7.93 (d, 1H), 7.83 (d, 1H), 7.72 (d, 1H), 7.65 (dd, 3H), 7.49 (q, 2H), 7.41 (t, 2H), 7.36 (t, 4H), 7.32 (d, 2H), 7.28 (d, 2H), 7.23 (d, 2H), 6.82 (s, 1H), 6.72 (d, 3H), 5.79 (s, 2H), 5.63 (s, 2H) ppm; **^{13}C NMR** (100 MHz, DMSO-*d6*): δ = 148.89, 142.03, 140.70, 135.19, 135.09, 131.32, 129.55, 129.48, 128.76, 128.48, 127.09, 125.82, 125.38, 123.87, 123.70, 123.40, 114.07, 113.99, 55.37, 49.71, 49.29 ppm; HRMS(ESI): m/z calc. for $[C_{39}H_{30}N_3O_2S_2]^+$ 636.1774, found 636.1783 $[M\text{-}ClO_4]^+$.

Synthesis of **C2-CF$_3$**

C2-CF$_3$ was synthesized according to the synthetic procedure of **C2-H**. The product obtained was a blue powder (103 mg, 51.6 % yield). **^{1}H NMR** (400 MHz, DMSO-*d6*): δ = 8.03 (d, 2H), 7.95 (d, 1H), 7.87 (d, 1H), 7.73 (d, 1H), 7.65 (d, 1H),

7.57 (d, 2H), 7.50 (q, 2H), 7.40 (d, 2H), 7.36 (d, 3H), 7.27 (d, 2H), 6.75 (m, 4H), 5.80 (s, 2H), 5.64 (s, 2H) ppm; **^{13}C NMR** (100 MHz, DMSO-*d6*): δ = 142.01, 140.67, 135.20, 135.11, 130.60, 130.12, 129.63, 129.55, 129.48, 129.30, 128.85, 128.77, 128.64, 128.48, 127.08, 125.79, 125.71, 125.53, 123.80, 123.41, 114.05, 113.96, 49.71, 49.24 ppm; HRMS(ESI): m/z calc. for $[C_{40}H_{30}F_3N_2S_2]^+$ 659.1797, found 659.1802 $[M\text{-}ClO_4]^+$.

Synthesis of **C2-COOH**

C2-COOH was synthesized according to the synthetic procedure of **C2-H**. The product obtained was a blue powder (107 mg, 49.2 % yield). **^{1}H NMR** (400 MHz, DMSO-*d6*): δ = 8.22 (d, 2H), 7.90 (d, 1H), 7.86 (d, 1H), 7.72 (d, 1H), 7.62 (d, 1H), 7.51 (d, 1H), 7.47 (d, 1H), 7.43 (d, 2H), 7.40 (d, 2H), 7.34 (m, 6H), 7.27 (d, 2H), 7.23 (d, 2H), 6.84 (s, 1H), 6.71 (s, 1H), 6.69 (s, 1H), 6.62 (s, 1H), 5.80 (s, 2H), 5.61 (s, 2H) ppm; **^{13}C NMR** (100 MHz, DMSO-*d6*): δ = 142.07, 140.64, 135.21, 135.16, 131.20, 129.78, 129.55, 129.47, 127.77, 127.49, 127.09, 125.09, 125.52, 123.72, 123.78, 114.04, 113.77, 49.758, 49.15, 34.81, 31.42, 22.53, 14.43 ppm; HRMS(ESI): m/z calc. for $[C_{40}H_{31}O_2N_2S_2]^+$ 635.1821, found 635.1818 $[M\text{-}ClO_4]^+$.

Synthesis of **C2-OMe**

C2-OMe was synthesized according to the synthetic procedure of **C2-H**. The product obtained was a blue powder (94 mg, 44.1 % yield). **^{1}H NMR** (400 MHz, DMSO-*d6*): δ = 7.88 (t, 2H), 7.73 (d, 1H), 7.59 (d, 1H), 7.51 (d, 1H), 7.44 (d, 1H), 7.40 (d, 2H), 7.36 (s, 3H), 7.32 (d, 2H), 7.27-7.22 (m, 9H), 6.82 (s, 1H), 6.68 (s, 1H), 6.65 (s, 1H), 6.56 (s, 1H), 5.80 (s, 2H), 5.58 (s, 2H), 3.93 (s, 3H) ppm; **^{13}C NMR** (100

MHz, DMSO-*d6*): δ = 161.26, 142.13, 140.61, 135.24, 135.22, 131.04, 129.55, 129.46, 128.70, 128.48, 128.41, 127.09, 127.06, 125.80, 125.30, 125.24, 123.62, 123.30, 115.89, 114.01, 113.55, 55.88, 49.82, 49.01 ppm; HRMS(ESI): m/z calc. for $[C_{40}H_{33}N_2OS_2]^+$ 621.2029, found 621.2025 $[M-ClO_4]^+$.

Synthesis of **C2-NH2**

C2-NH2 was synthesized according to the synthetic procedure of **C2-H**. Since the reaction uses an inorganic base, the Schiff base is hydrolyzed to obtain $C2-NH_2$ with a para-substituent of an amino group. The product obtained was a blue powder (88 mg, 40.7 % yield). **^{1}H NMR** (400 MHz, DMSO-*d6*): δ = 7.87 (t, 2H), 7.71 (t, 1H), 7.52 (dt, 2H), 7.56-7.32 (ddq, 9H), 7.30 -7.22 (t, 6H), 6.91 (t, 2H), 6.76 (d, 2H), 6.57 (dd, 1H), 6.53 (s, 1H), 5.77 (m, 2H), 5.54 (s, 2H) ppm; **^{13}C NMR** (100 MHz, DMSO-*d6*): δ = 142.18, 140.63, 135.31, 135.24, 131.05, 13.012, 129.55, 129.45, 128.59, 128.44, 128.37, 127.11, 127.04, 125.69, 125.11, 125.06, 123.55, 123.21, 115.04, 113.86, 113.32, 99.85, 49.79, 48.89 ppm; HRMS(ESI): m/z calc. for $[C_{39}H_{32}N_3S_2]^+$ 606.2032, found 606.2023 $[M-ClO_4]^+$.

4. Supplementary Content

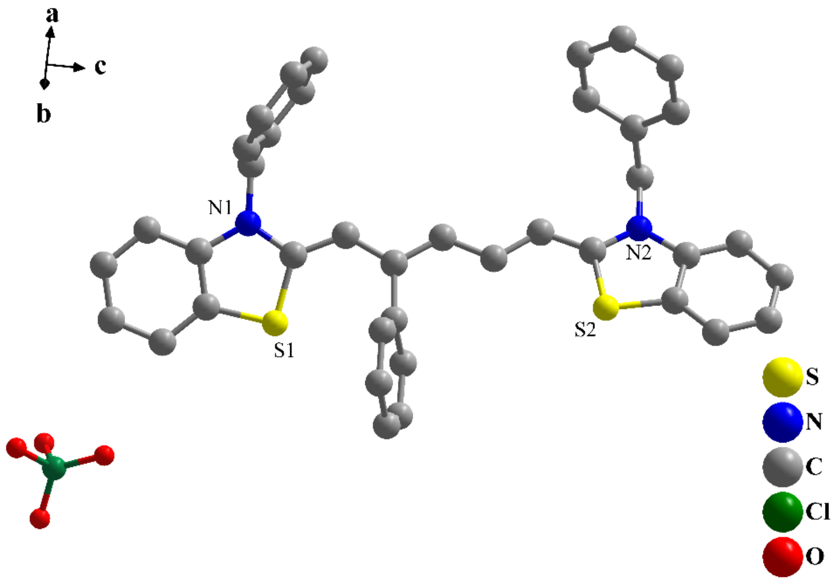

Figure S1. Crystal structures of **C2-H**. All H atoms are omitted for clarity atomic scheme, S: yellow, N: blue, C: gray, Cl: green, O: red.

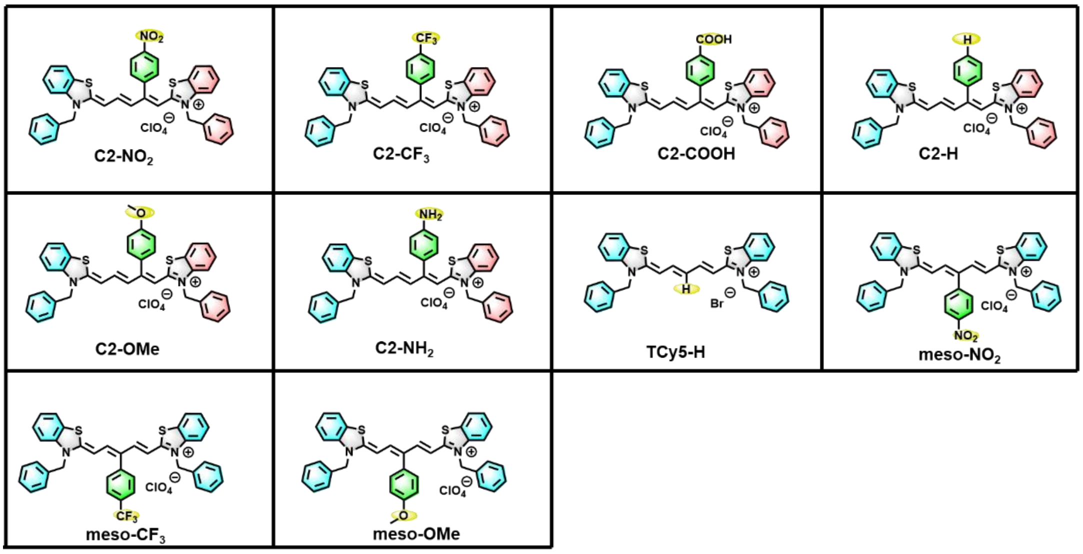

Figure S2. Chemical structural of different dyes

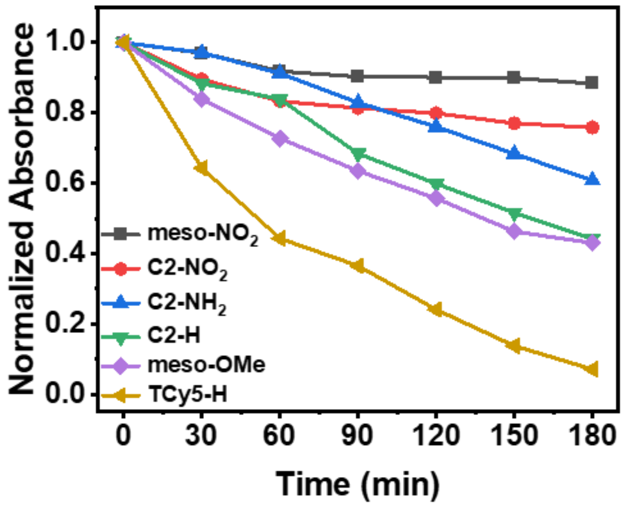

Figure S3. Photostabilities of TCy5-H, C2-NO_2, C2-H, C2-NH_2, meso-NO_2 and meso-OMe under 660 nm light irradiation.

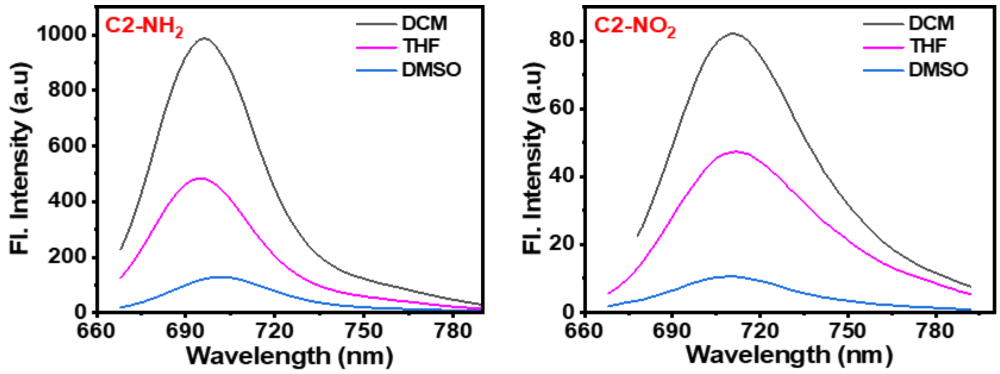

Figure S4. Fluorescence intensities spectrum of **C2-NO_2** and **C2-NH_2** polarity of the solvents

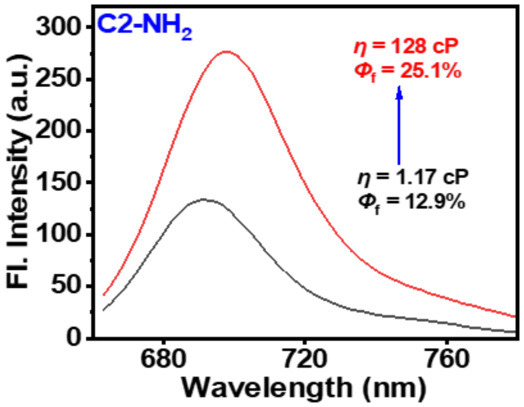

Figure S5. Fluorescence intensities spectrum of **C2-NO_2** and **C2-NH_2** in different viscosity solvents

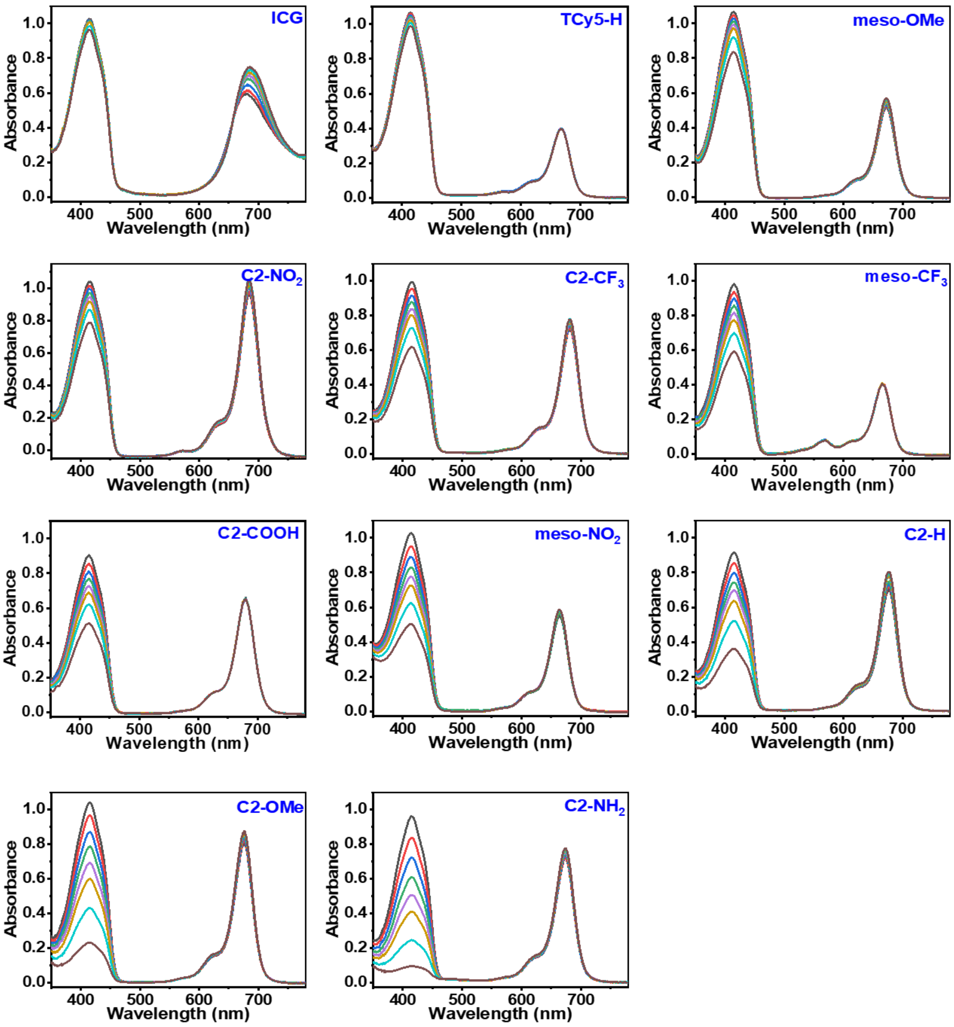

Figure S6. DPBF degradation induced by **Cyanine dyes** under 660 nm light irradiation.

Table S1: Calculated data of relative singlet oxygen yield.

**Compd.**	**K**	**O.D.**	**F**	Φ_{Δ}^{rel}
ICG	0.00482	0.48	0.66887	1.0
C2-NO_2	0.02438	0.36	0.56348	6.0
C2-CF_3	0.03691	0.35	0.55332	9.3
C2-COOH	0.04155	0.33	0.53633	10.8
C2-H	0.05985	0.43	0.62846	13.2
C2-OMe	0.08058	0.50	0.68377	16.4
C2-NH_2	0.11991	0.50	0.68377	24.3
meso-NO_2	0.05582	0.50	0.70488	11.0
meso-CF_3	0.04146	0.38	0.57903	9.9
meso-OMe	0.01855	0.38	0.58121	4.4
TCy-H	0.00753	0.35	0.54918	2.3

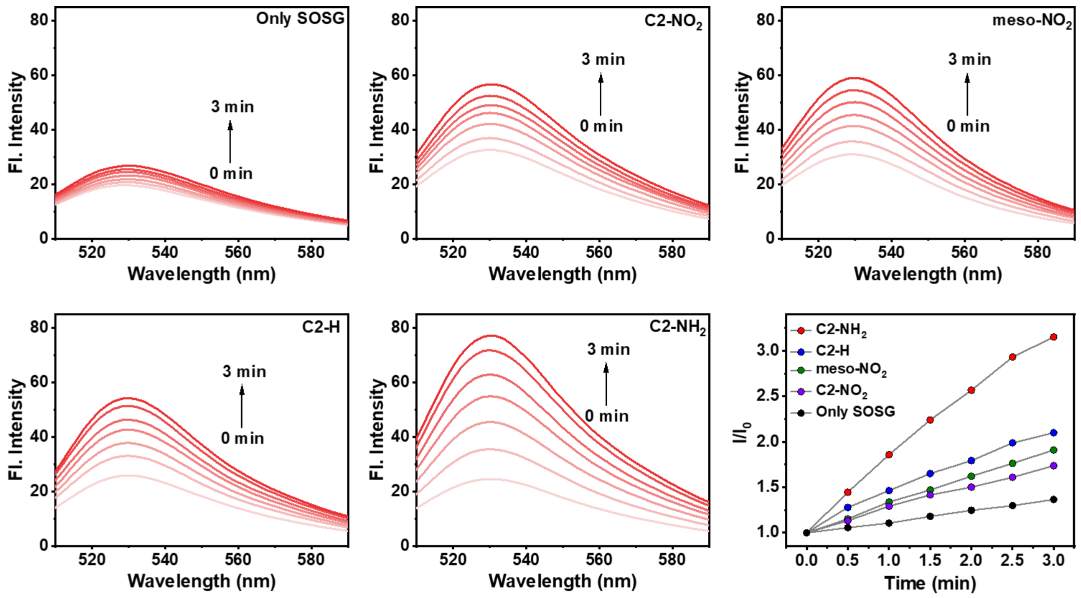

Figure S7. Fluorescence spectra for $^1O_2$ using **SOSG** as fluorescence probe under 660 nm light irradiation for 3 min in water with **Cyanine dyes**.

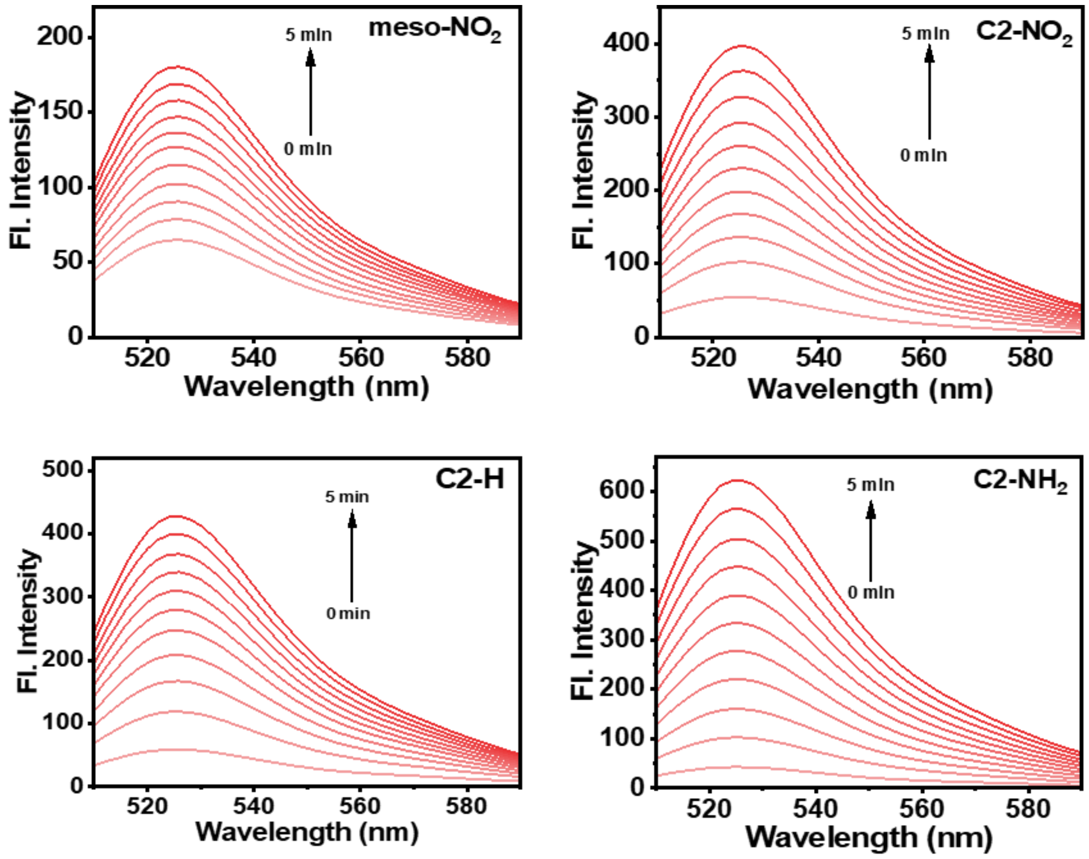

Figure S8. Fluorescence spectra for $O_2^{\bullet-}$ using **DHR123** as fluorescence probe under 660 nm light irradiation for 5 min in water with **Cyanine dyes**.

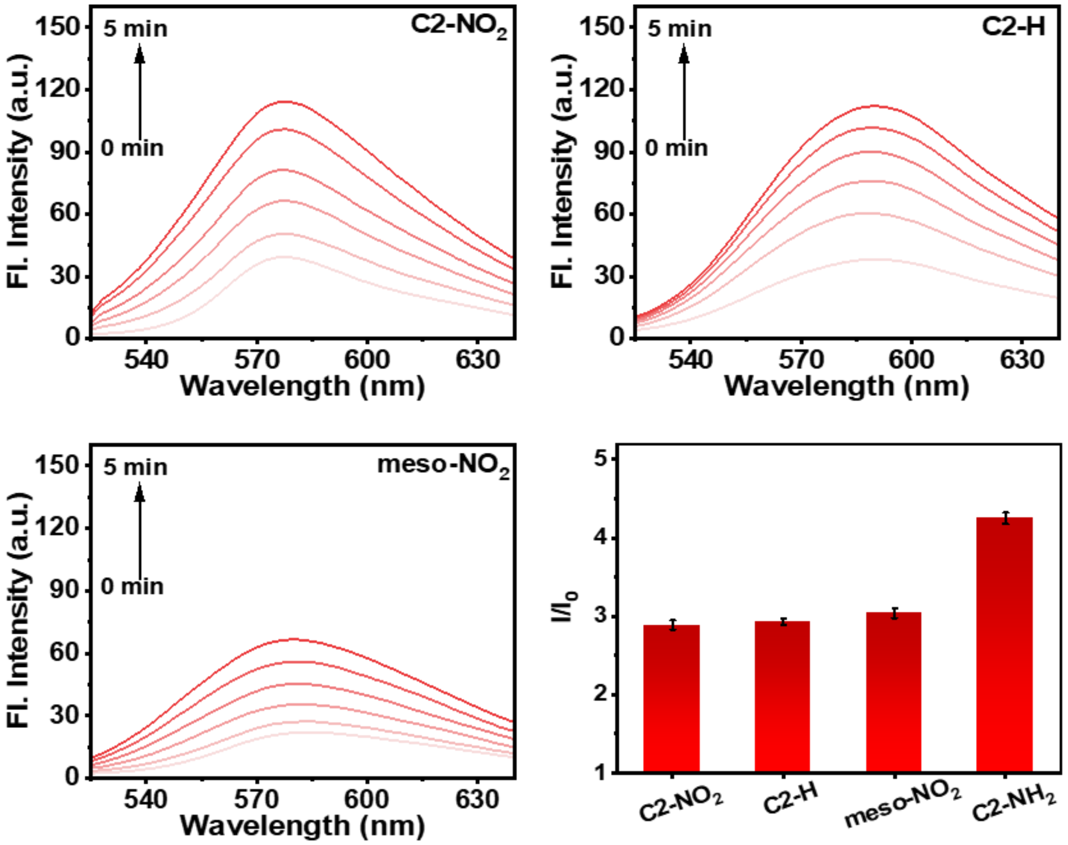

Figure S9. Fluorescence spectra for $O_2^{\bullet-}$ using **DHE** as fluorescence probe under 660 nm light irradiation for 5 min in water with **Cyanine dyes**.

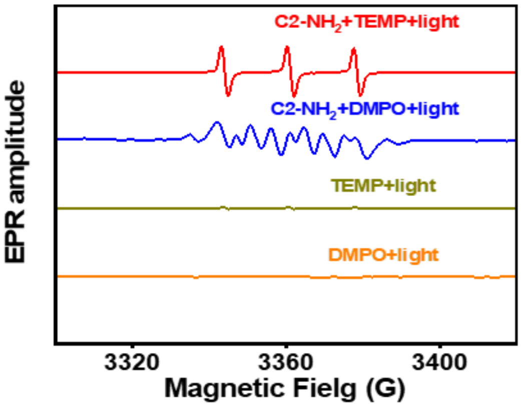

Figure S10. electron spin-resonance spectroscopy (ESR) signals of the mixture containing **C2-NH**$_2$ and TEMP or DMPO under 660 nm light irradiation and the reference signal.

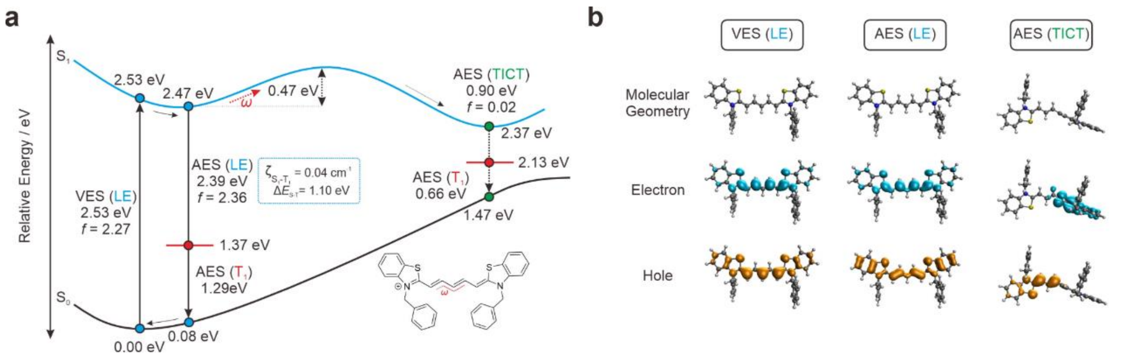

Figure S11. (a) Schematic illustration of the TICT mechanism and the excitation/de-excitation energy along with oscillator strength (f) of the key states during this process. (b) Optimized molecular structures of **TCy5-H** in the ground and excited states, as well as the corresponding electron and hole distributions in dichloromethane. VES and AES denote a vertically excited state and an adiabatic excited state, respectively.

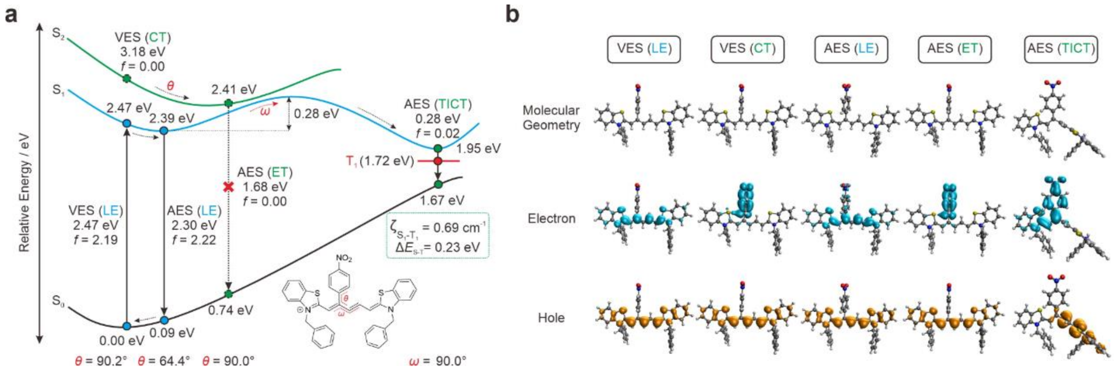

Figure S12. (a) Schematic illustration of the TICT and PET mechanisms and the excitation/de-excitation energy along with oscillator strength (f) of the key states during this process. (b) Optimized molecular structures of **C2-NO2** in the ground and excited states, as well as the corresponding electron and hole distributions in dichloromethane. VES and AES denote a vertically excited state and an adiabatic excited state, respectively.

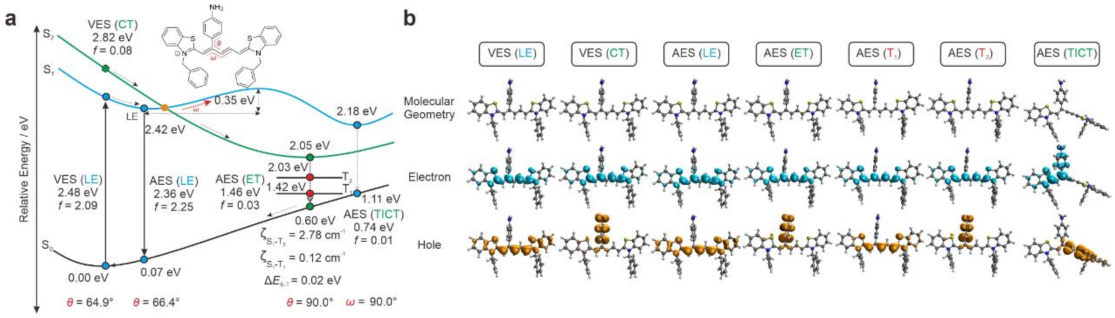

Figure S13. (a) Schematic illustration of the TICT and PET mechanisms and the excitation/de-excitation energy along with oscillator strength (f) of the key states during this process. (b) Optimized molecular structures of **C2-NH2** in the ground and excited states, as well as the corresponding electron and hole distributions in dichloromethane. VES and AES denote a vertically excited state and an adiabatic excited state, respectively.

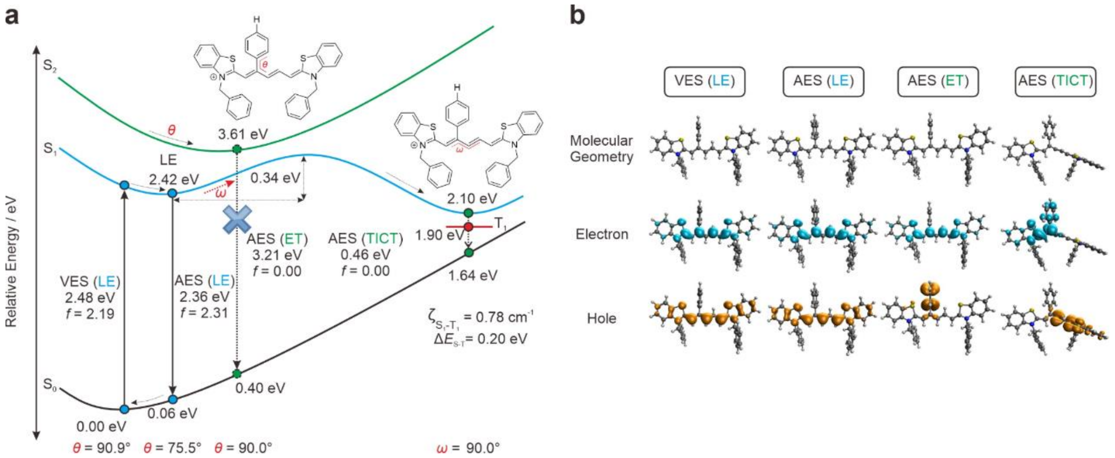

Figure S14. (a) Schematic illustration of the TICT and PET mechanisms and the excitation/de-excitation energy along with oscillator strength (f) of the key states during this process. (b) Optimized molecular structures of **C2-H** in the ground and excited states, as well as the corresponding electron and hole distributions in dichloromethane. VES and AES denote a vertically excited state and an adiabatic excited state, respectively.

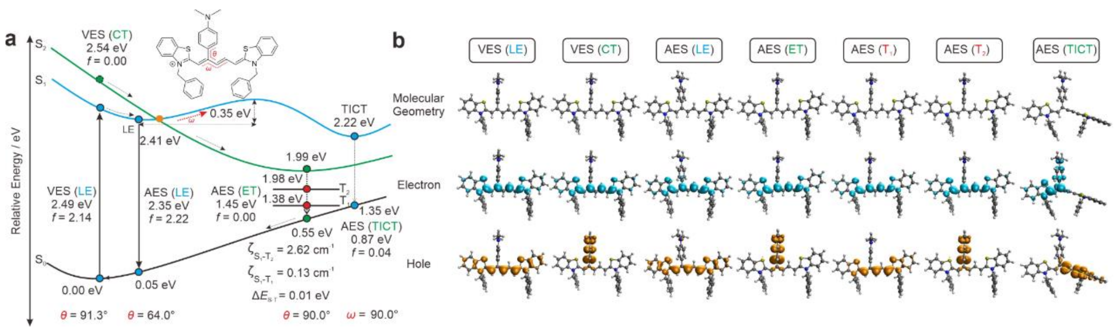

Figure S15. (a) Schematic illustration of the TICT and PET mechanisms and the excitation/de-excitation energy along with oscillator strength (f) of the key states during this process. (b) Optimized molecular structures of **C2-NMe2** in the ground and excited states, as well as the corresponding electron and hole distributions in dichloromethane. VES and AES denote a vertically excited state and an adiabatic excited state, respectively.

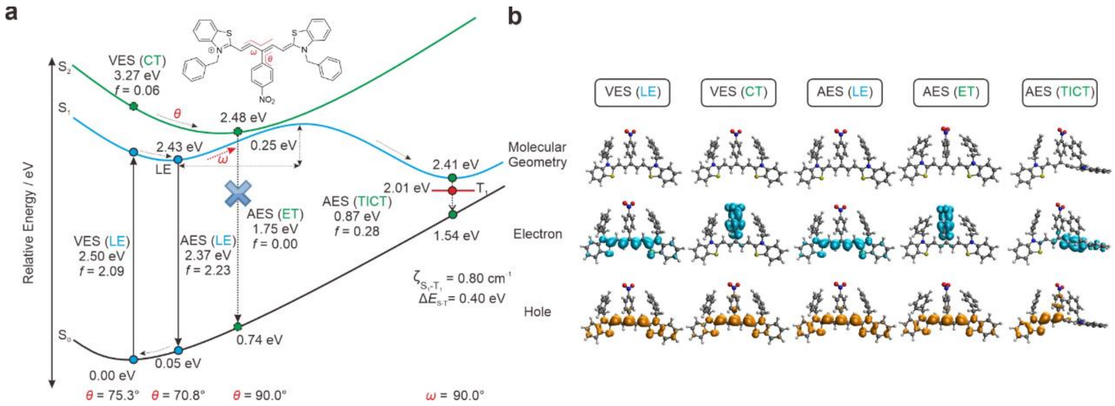

Figure S16. (a) Schematic illustration of the TICT and PET mechanisms and the excitation/de-excitation energy along with oscillator strength (f) of the key states during this process. (b) Optimized molecular structures of **meso-NO2** in the ground and excited states, as well as the corresponding electron and hole distributions in dichloromethane. VES and AES denote a vertically excited state and an adiabatic excited state, respectively.

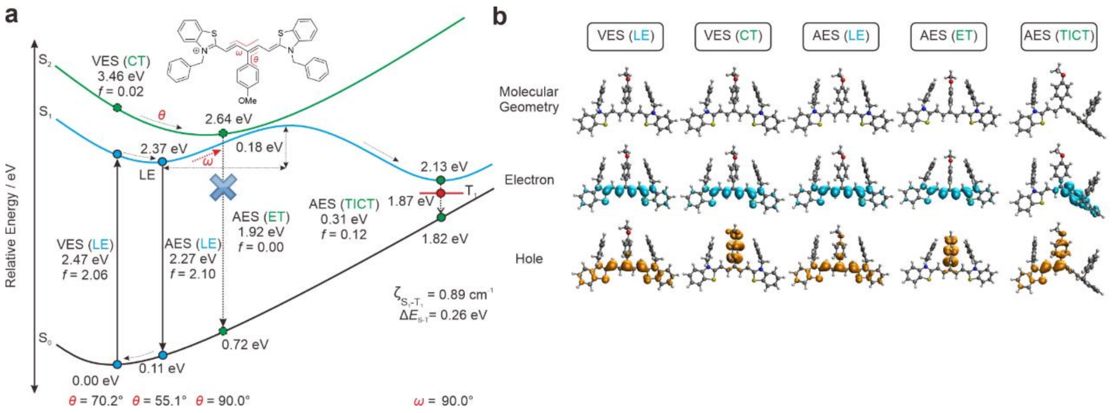

Figure S17. (a) Schematic illustration of the TICT and PET mechanisms and the excitation/de-excitation energy along with oscillator strength (f) of the key states during this process. (b) Optimized molecular structures of **meso-OMe** in the ground and excited states, as well as the corresponding electron and hole distributions in dichloromethane. VES and AES denote a vertically excited state and an adiabatic excited state, respectively.

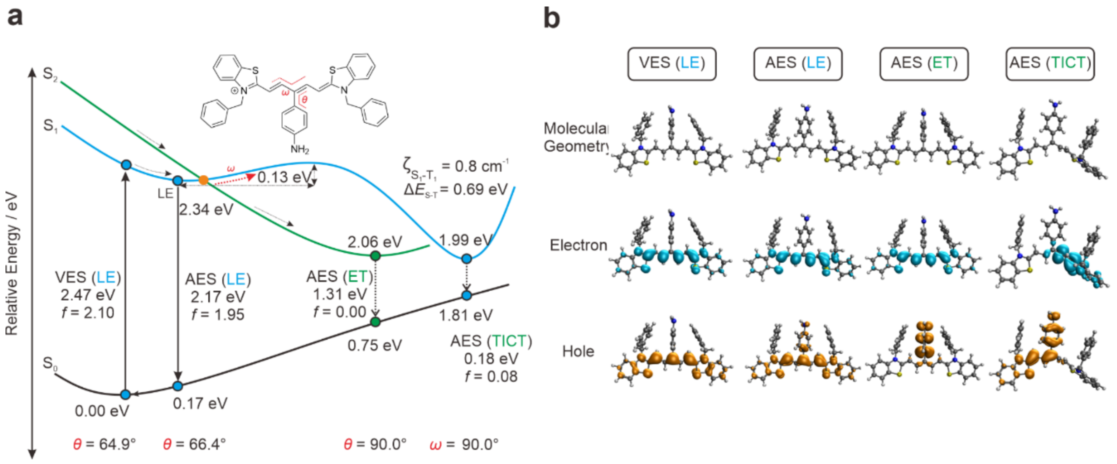

Figure S18. (a) Schematic illustration of the TICT and PET mechanisms and the excitation/de-excitation energy along with oscillator strength (f) of the key states during this process. (b) Optimized molecular structures of **meso-NH2** in the ground and excited states, as well as the corresponding electron and hole distributions in dichloromethane. VES and AES denote a vertically excited state and an adiabatic excited state, respectively.

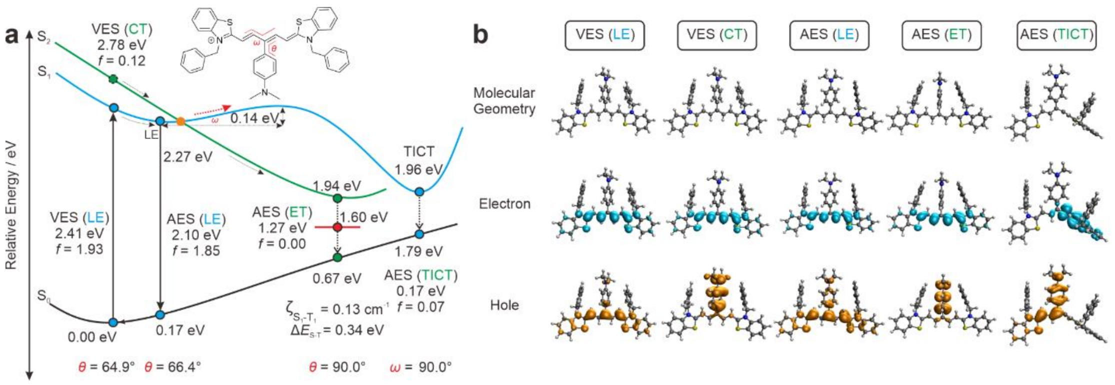

Figure S19. (a) Schematic illustration of the TICT and PET mechanisms and the excitation/de-excitation energy along with oscillator strength (f) of the key states during this process. (b) Optimized molecular structures of **meso-NMe₂** in the ground and excited states, as well as the corresponding electron and hole distributions in dichloromethane. VES and AES denote a vertically excited state and an adiabatic excited state, respectively.

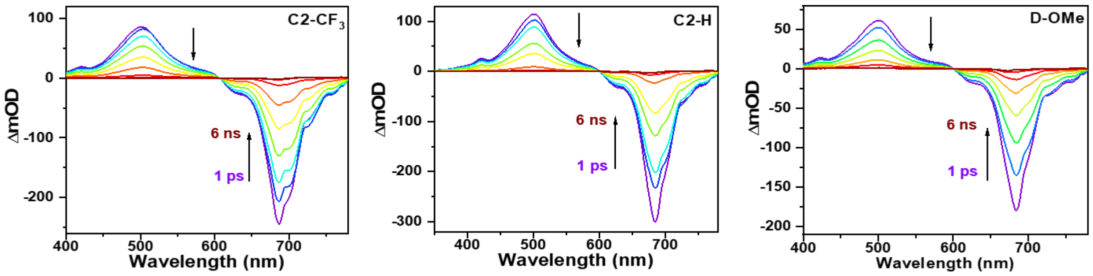

Figure S20. Nanosecond transient absorption spectra of **C2'-modification Cyanine dyes**.

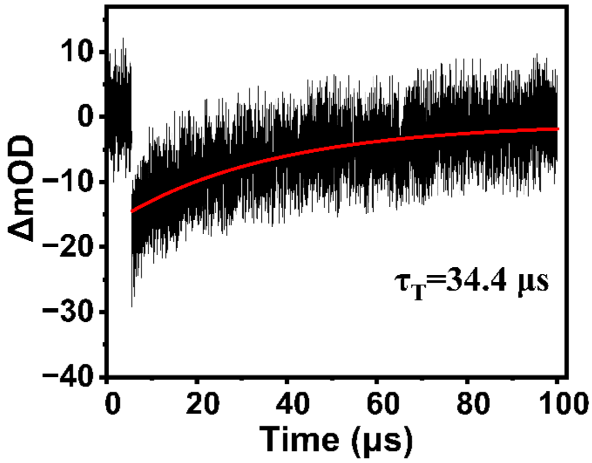

Figure S21. Triplet lifetime of **C2-OMe** tested by Flash Photolysis Spectrophotometer.

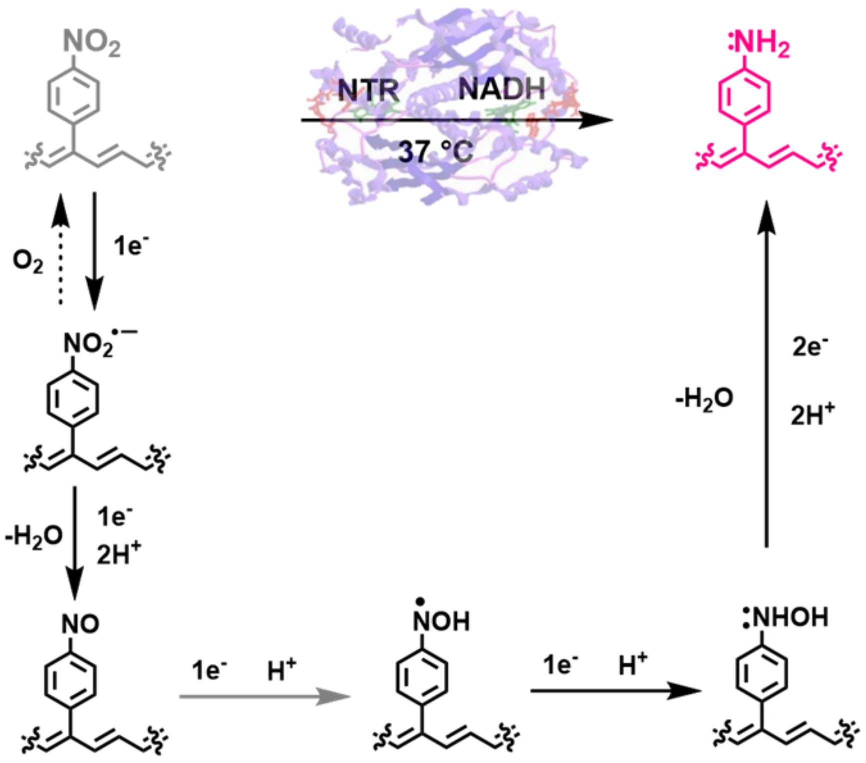

Figure S22. Deduced reactions mechanism of C2-NO_2 after sensing to NTR.

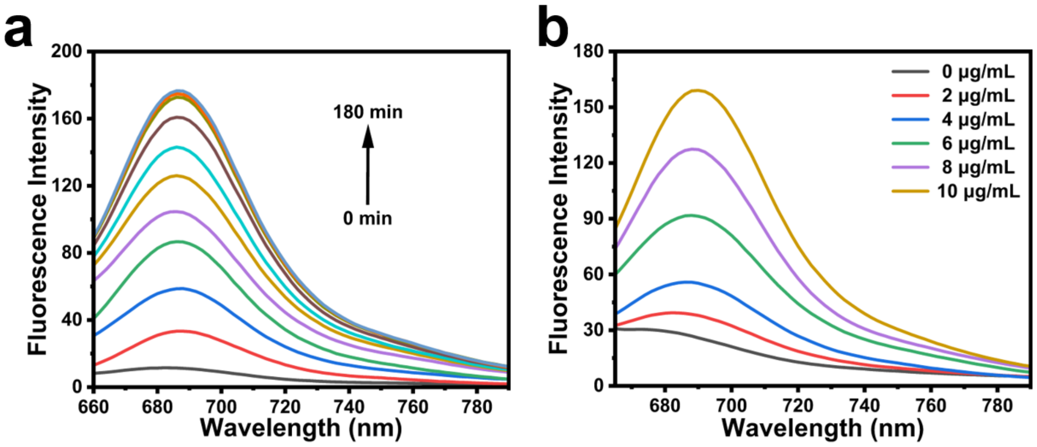

Figure S23. (a) Fluorescence spectra of **C2-NO_2** (10 μM) treated with NTR (10 μg mL^{-1}) and NADH (500 μM) as a function of time (0-180 min). (b) Fluorescence spectra of **C2-NO_2** (10 μM) treated with different concentrations of NTR (0-10 ug mL^{-1}) for 120 min.

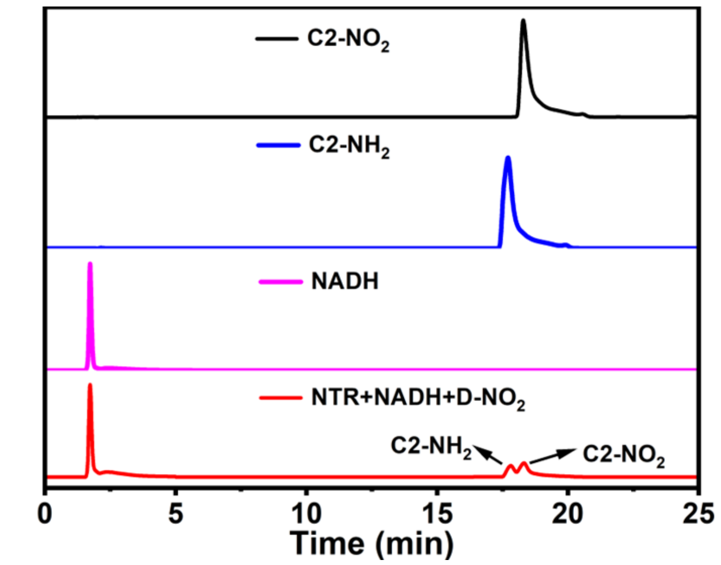

Figure S24. HPLC elution profiles of **C2-NO2** (black), **C2-NH2** (blue), NADH (purple) and the mixed solution (red) of **C2-NO2**, NTR (10 μg mL^{-1}) and NADH (500 μM).

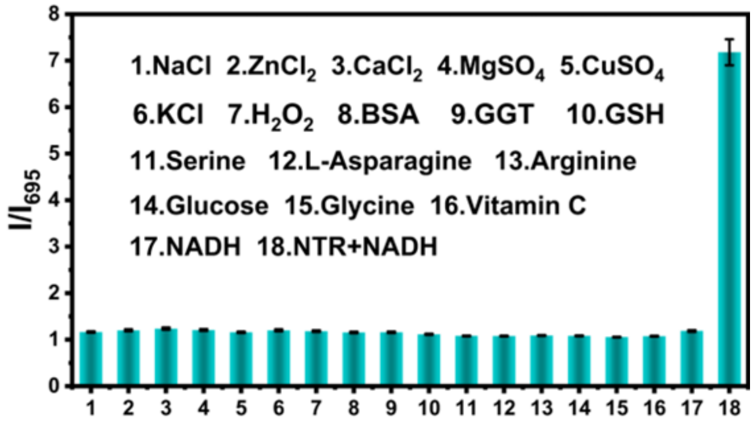

Figure S25. Fluorescence responses at λ_{em} = 695 nm of **C2-NO2** toward various biospecies.

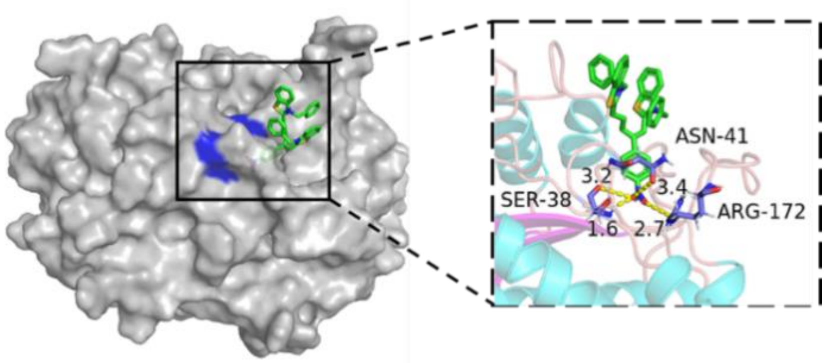

Figure S26. Molecular docking of **C2-NO2** in NTR pocket.

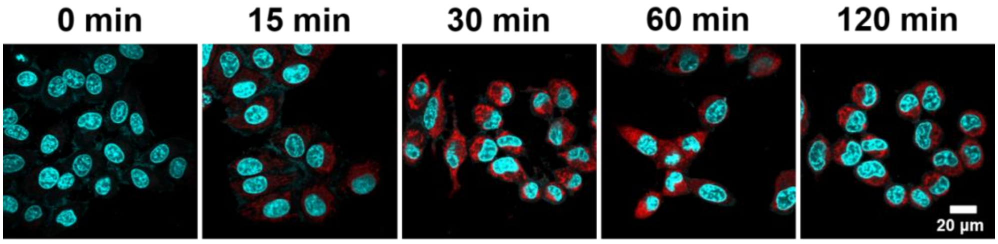

Figure S27. Cellular uptake images of **C2-NO2** in HepG2 cells under normoxia.

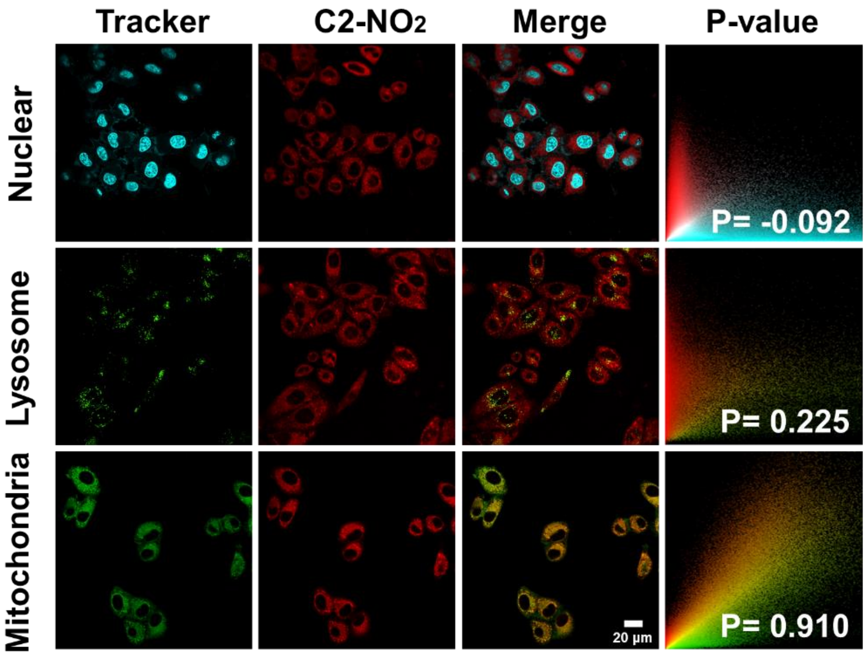

Figure S28. Subcellular colocalization images of **C2-NO2** (0.5 μM) and commercial trackersin HepG2 cells.

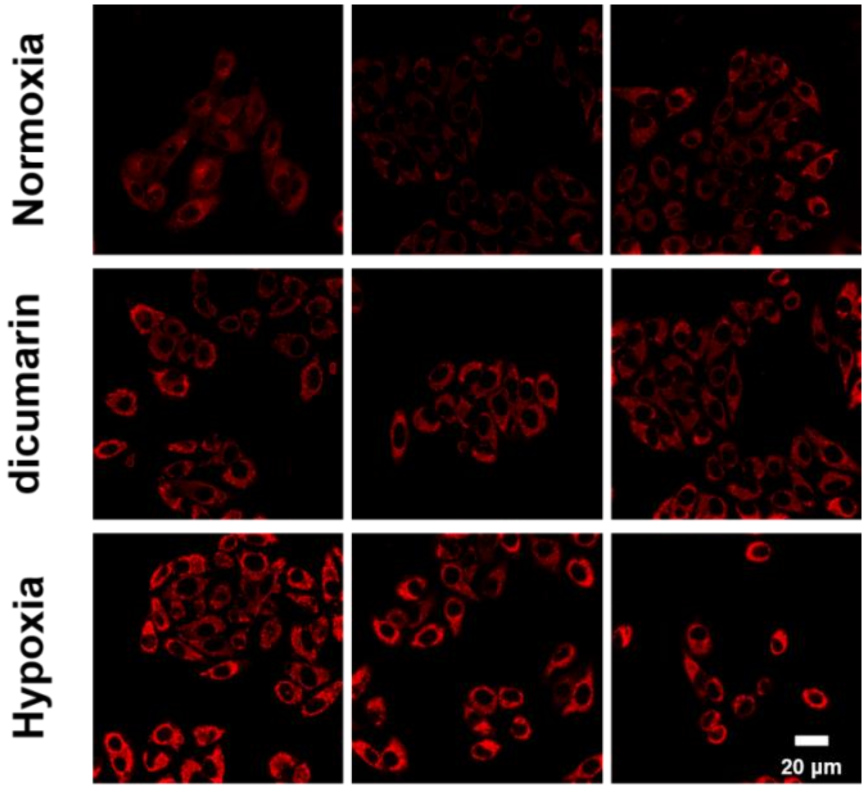

Figure S29. Confocal luminescence imaging of HepG2 cells incubated with **C2-NO2** (0.5 μM) under normoxia, hypoxia and hypoxia with dicumarin (0.1 mM) pretreating (λ_{ex}: 640 nm, λ_{em}: 690-740 nm).

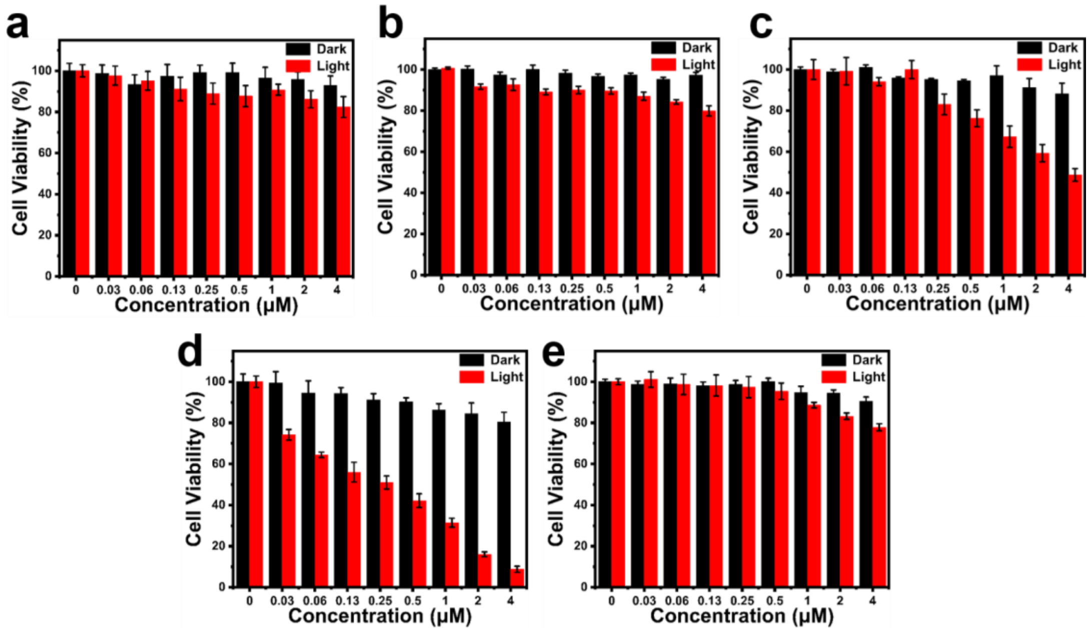

Figure S30. Cell viability of HepG2 cells under normoxia subjected to a range of (a) **ICG**, (b) **TCy5-H**, (c) **meso-NO2**, (d) **C2-NH2** and (e) **C2-NO2** concentrations under 20 mW cm^{-2} light irradiation for 15 min.

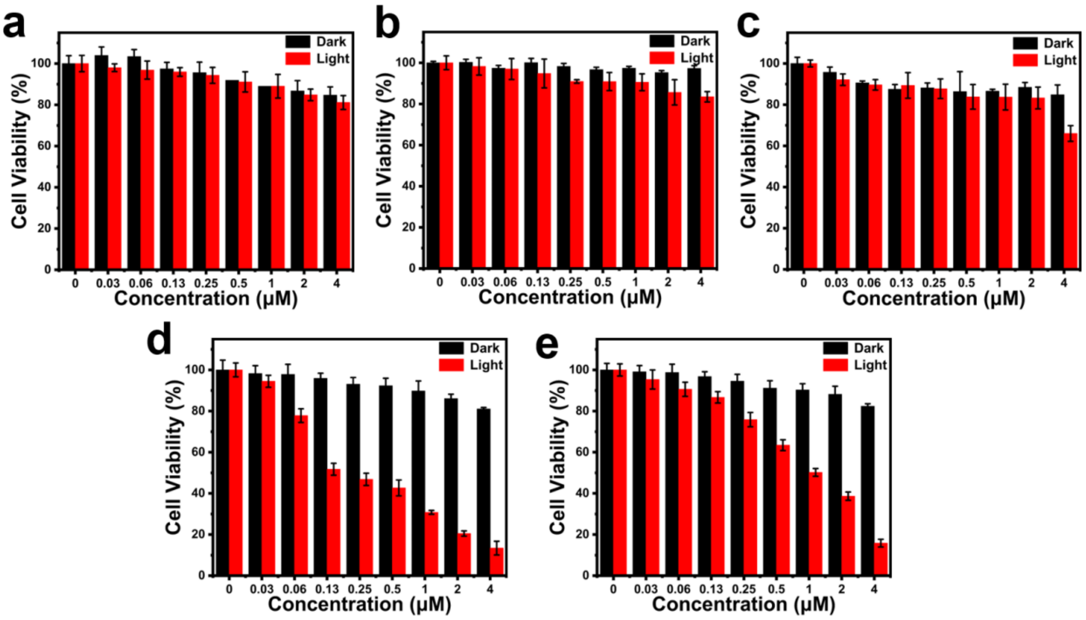

Figure S31. Cell viability of HepG2 cells under hypoxia subjected to a range of (a) **ICG**, (b) **TCy5-H**, (c) **meso-NO2**, (d) **C2-NH2** and (e) **C2-NO2** concentrations under 20 mW cm^{-2} light irradiation for 15 min.

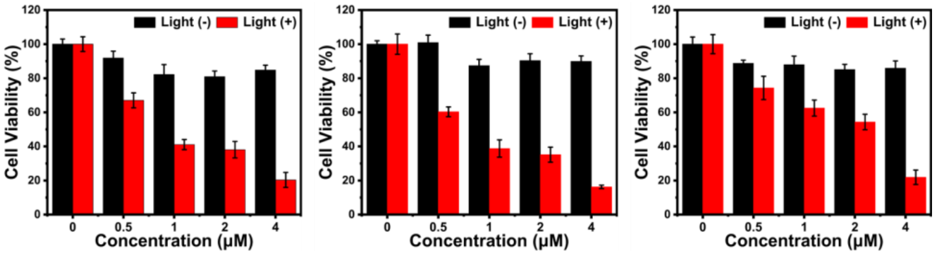

Figure S32. Cell viability of (a)4T1 cells, (b) A549 cells and (c) MCF7 cells under hypoxia subjected to a range of **C2-NO2** concentrations under 20 mW cm^{-2} light irradiation for 15 min.

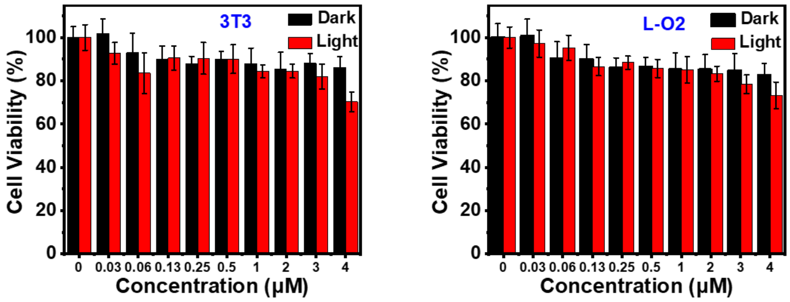

Figure S33. Cell viability of (a) 3T3 cells, (b) L-O2 cells under normoxia subjected to a range of **C2-NO2** concentrations under 20 mW cm^{-2} light irradiation for 15 min.

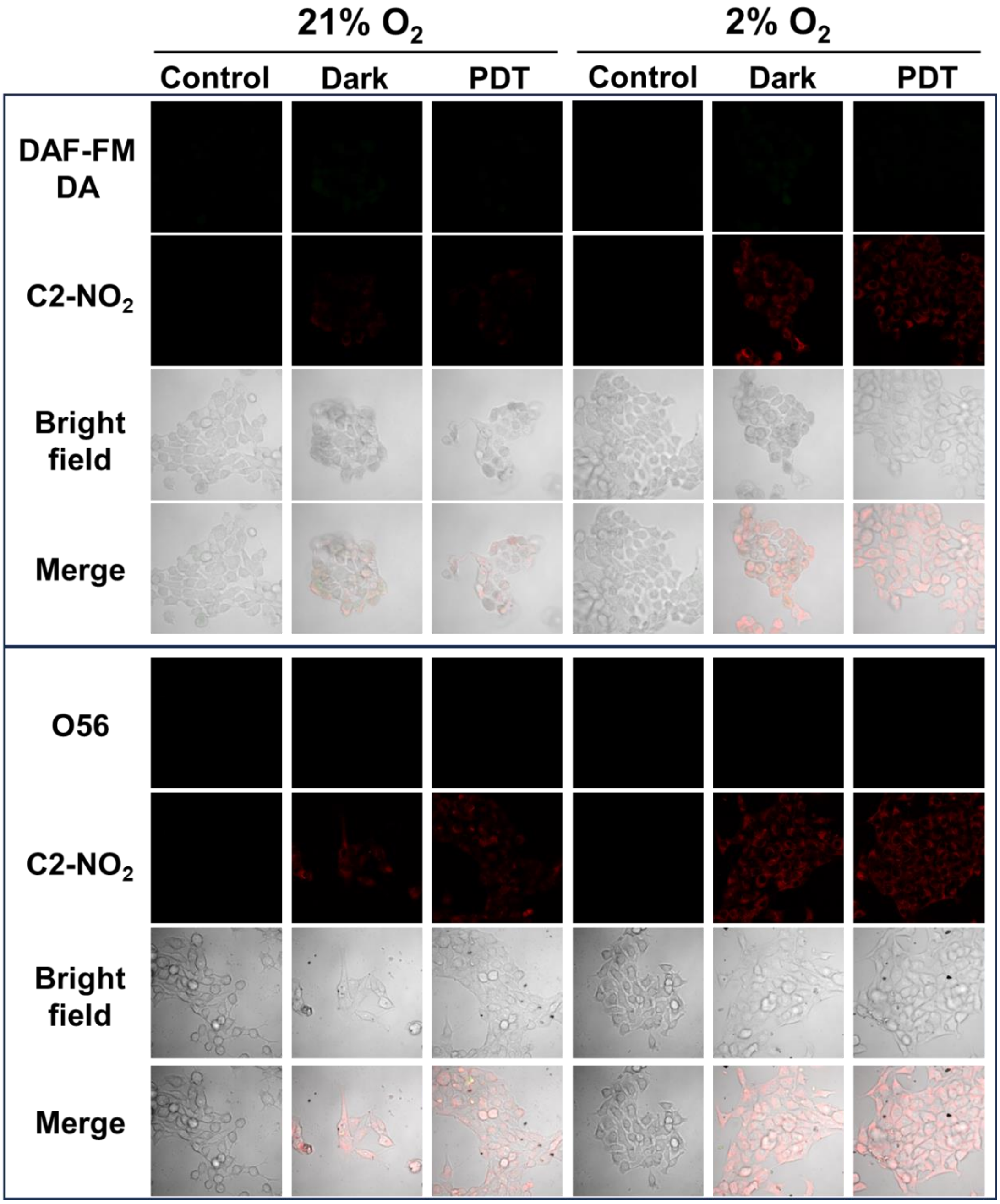

Figure S35. Intracellular NO and $ONOO^-$ production by C2-NO_2 in 4T1 cells monitored by the DAF-FMDA and O56 assays under different conditions., DAF-FMDA: λ_{ex}: 488 nm, λ_{em}: 510–560 nm; O56: λ_{ex}: 475 nm, λ_{em}: 510–560 nm.

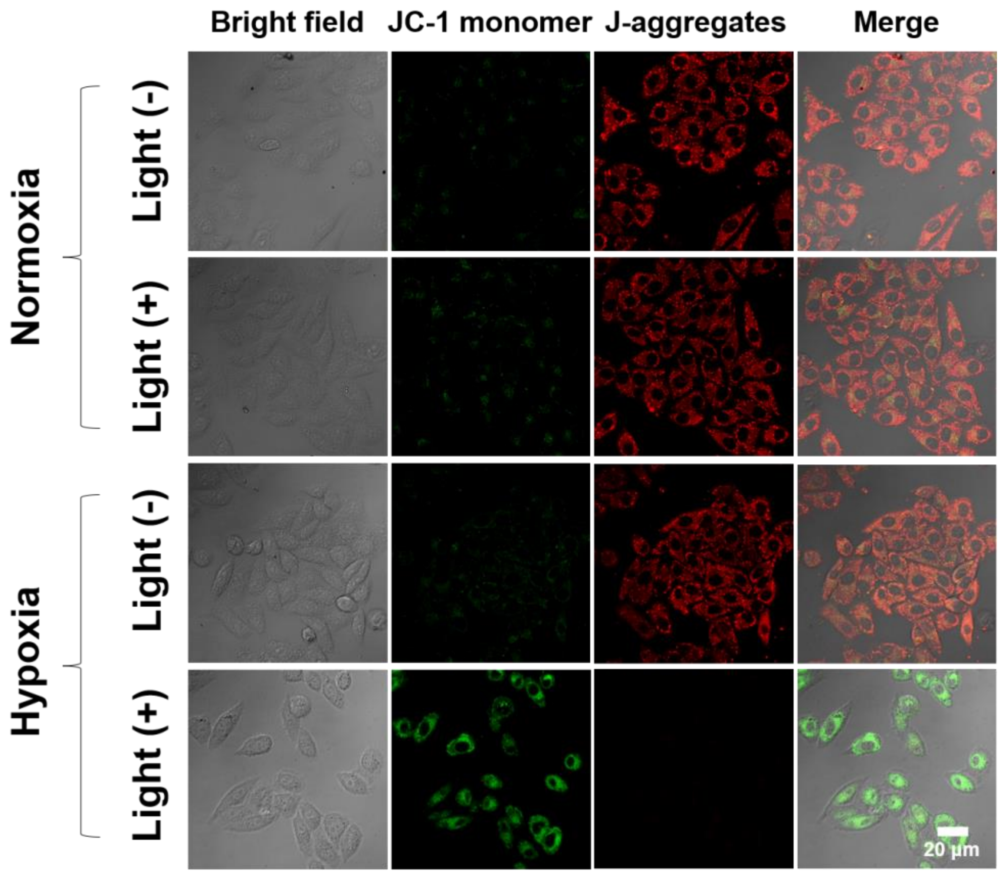

Figure S35. Mitochondrial membrane potential assay for **C2-NO2**-mediated photodamage of mitochondria under normoxia (10 mW cm^{-2}, 10 min) and hypoxia (10 mW cm^{-2}, 10 min), JC-1 monomer: λex: 488 nm, λem: 505–545 nm; JC-1 aggregate: λ_{ex}: 488 nm, λ_{em}: 560–590 nm.

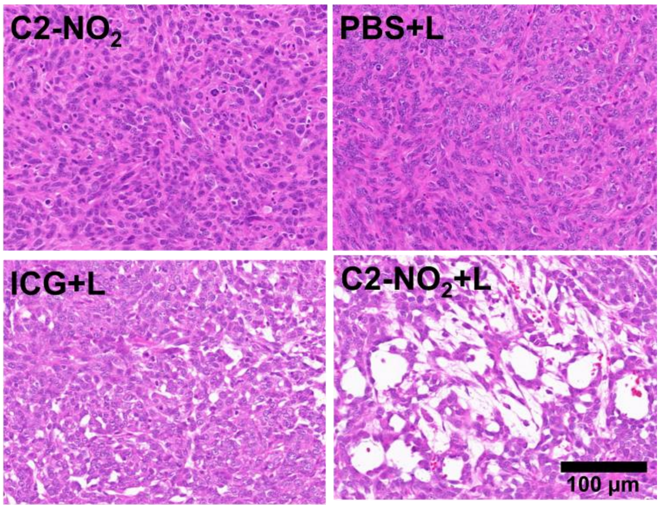

Figure S36. Hematoxylin and eosin (H&E)-stained tumors from Balb/C mice after different treatments.

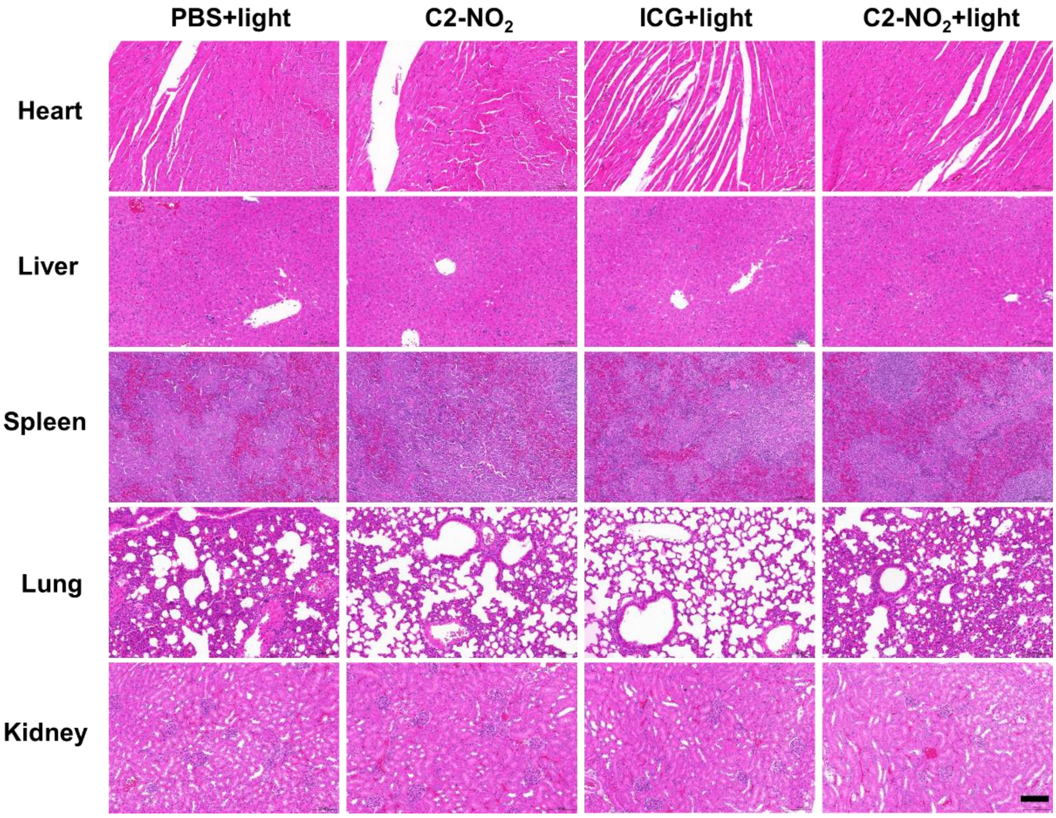

Figure S37. Typical images of H&E-stained heart, liver, spleen, lung, and kidney slices from mice 14 days post different treatments, scale bar = 100 μm.

5. ^{1}H NMR, ^{13}C NMR Spectrogram and HRMS Data

Compound 2

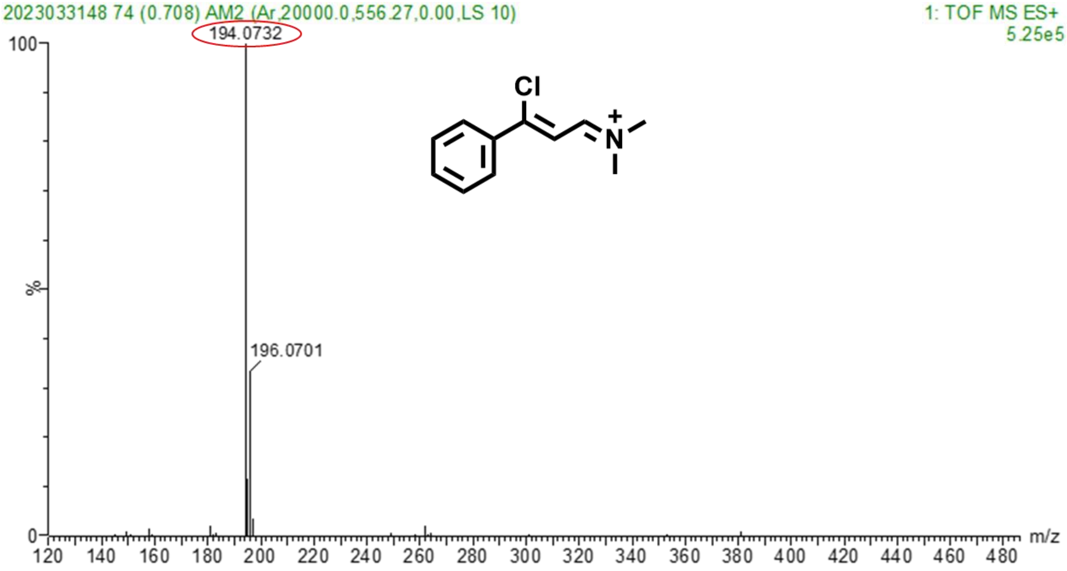

Figure S38. ESI-HRMS spectrum of Compound **2**.

C2-H

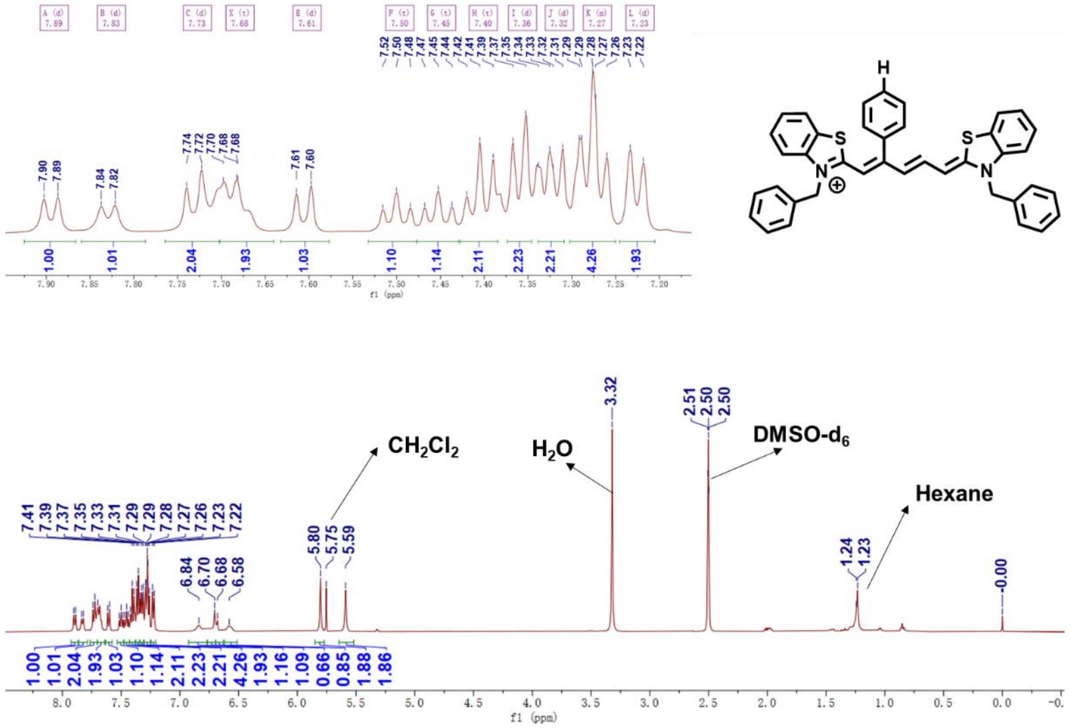

Figure S39. ^{1}H NMR spectrum of **C2-H**.

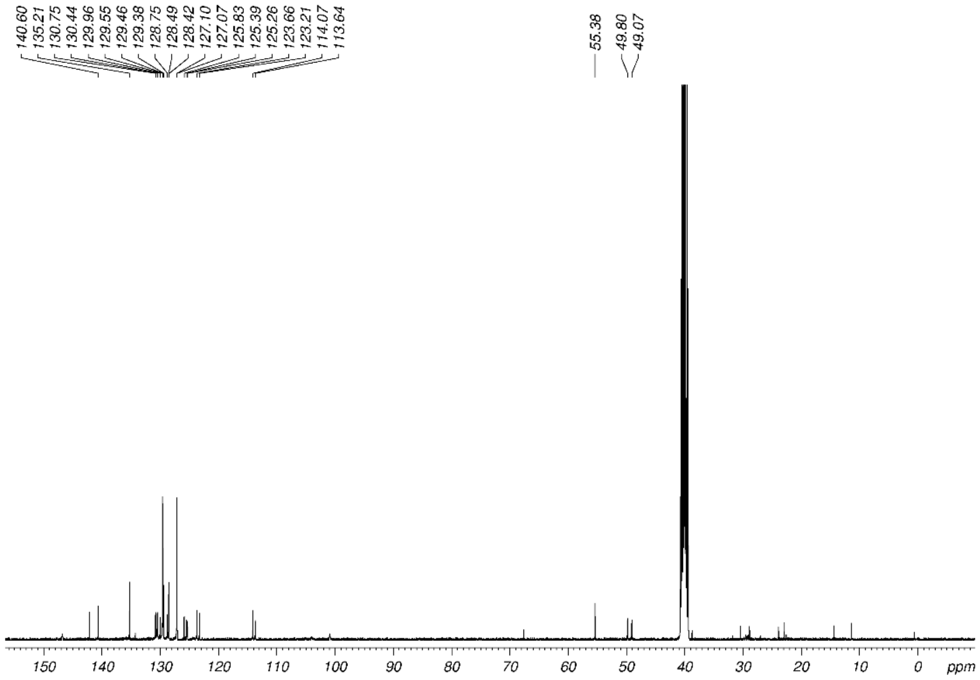

Figure S40. ^{13}C NMR spectrum of **C2-H**.

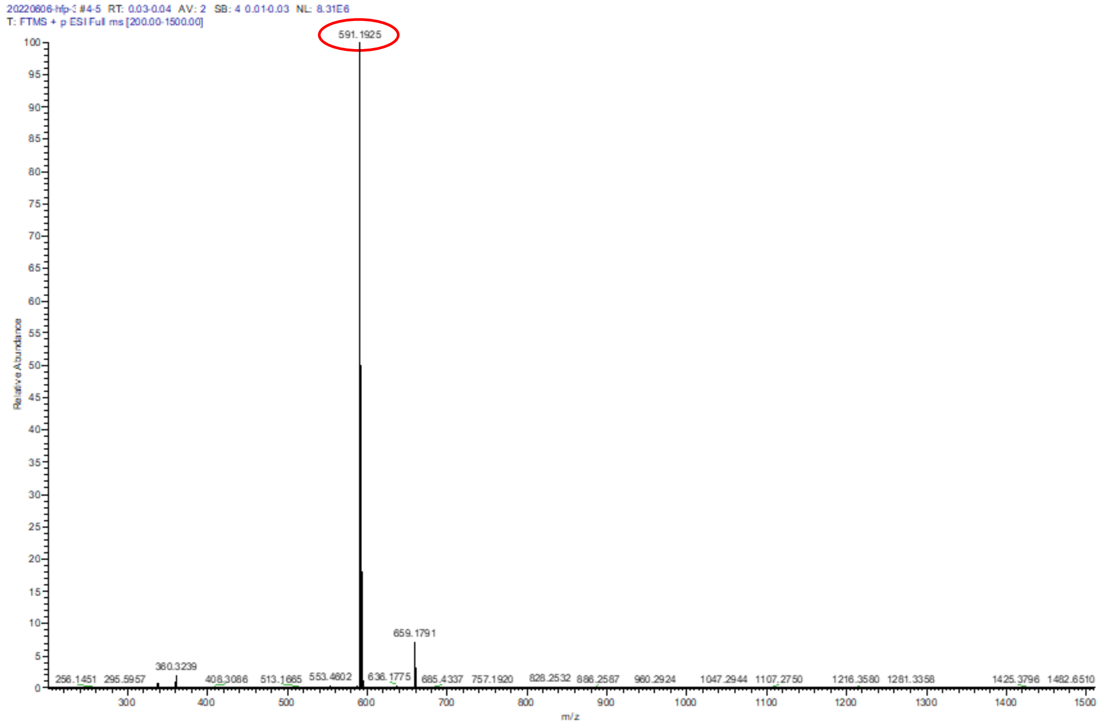

Figure S41. ESI-HRMS spectrum of **C2-H**.

Compound 3

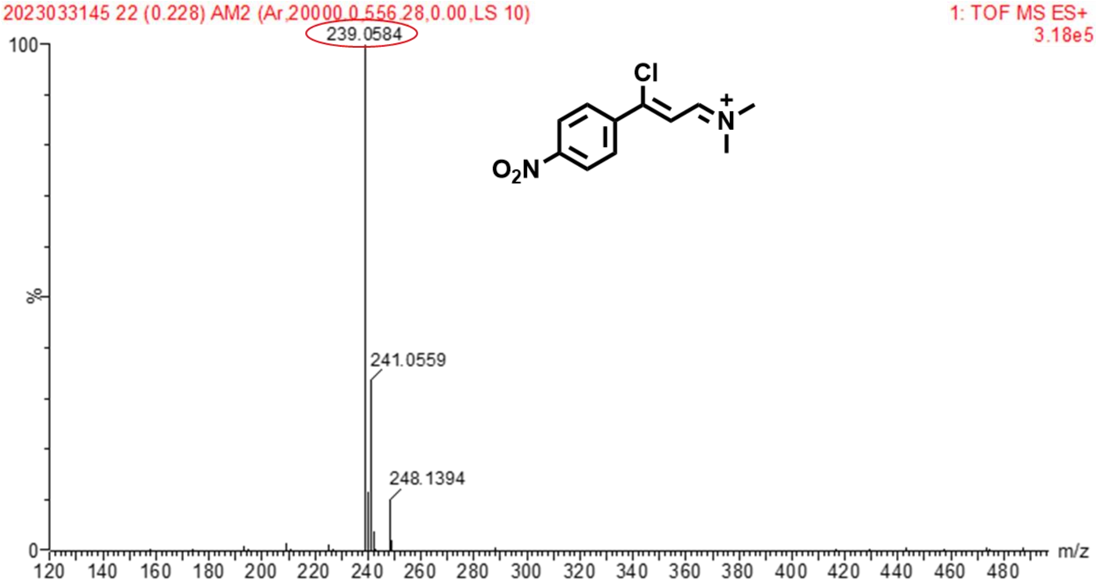

Figure S42. ESI-HRMS spectrum of Compound **3**.

C2-NO2

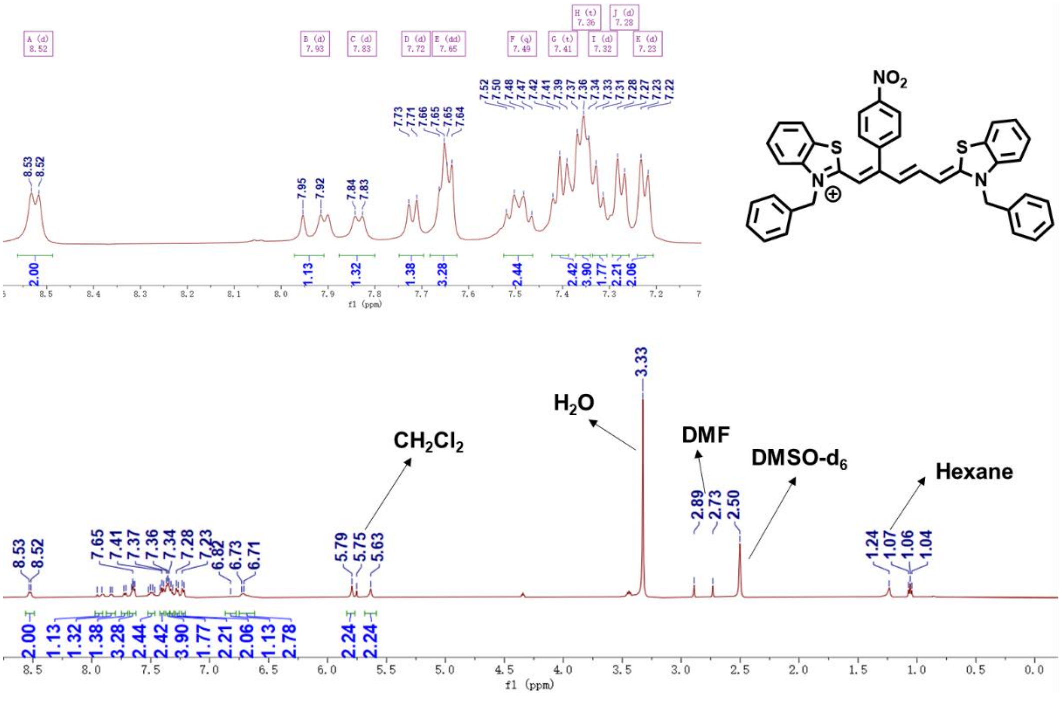

Figure S43. ^{1}H NMR spectrum of **C2-NO2**.

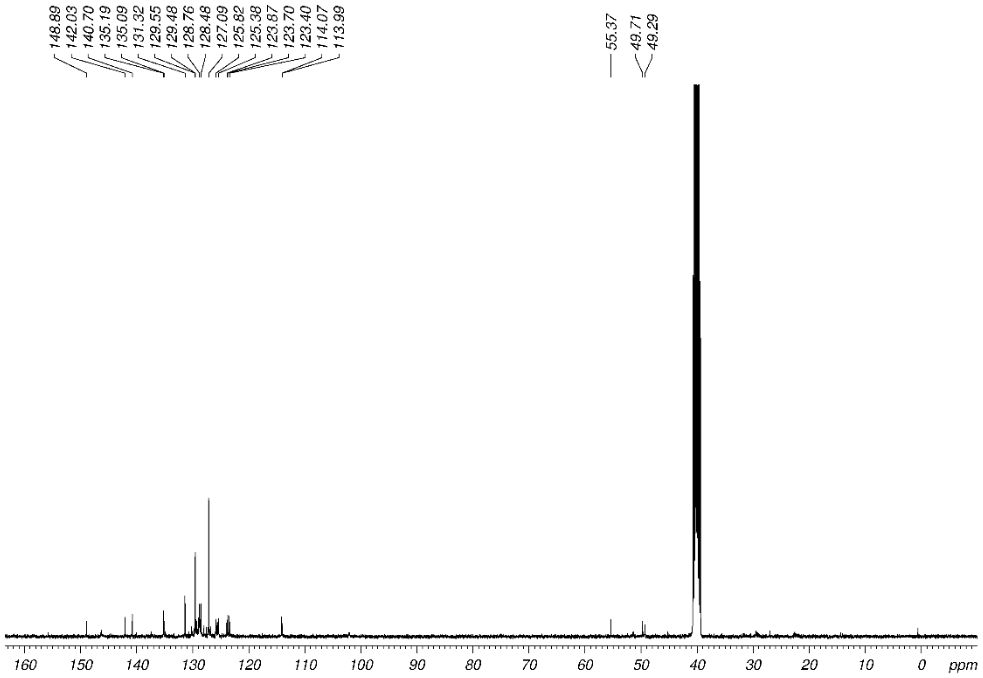

Figure S44. ^{13}C NMR spectrum of **C2-NO$_2$**.

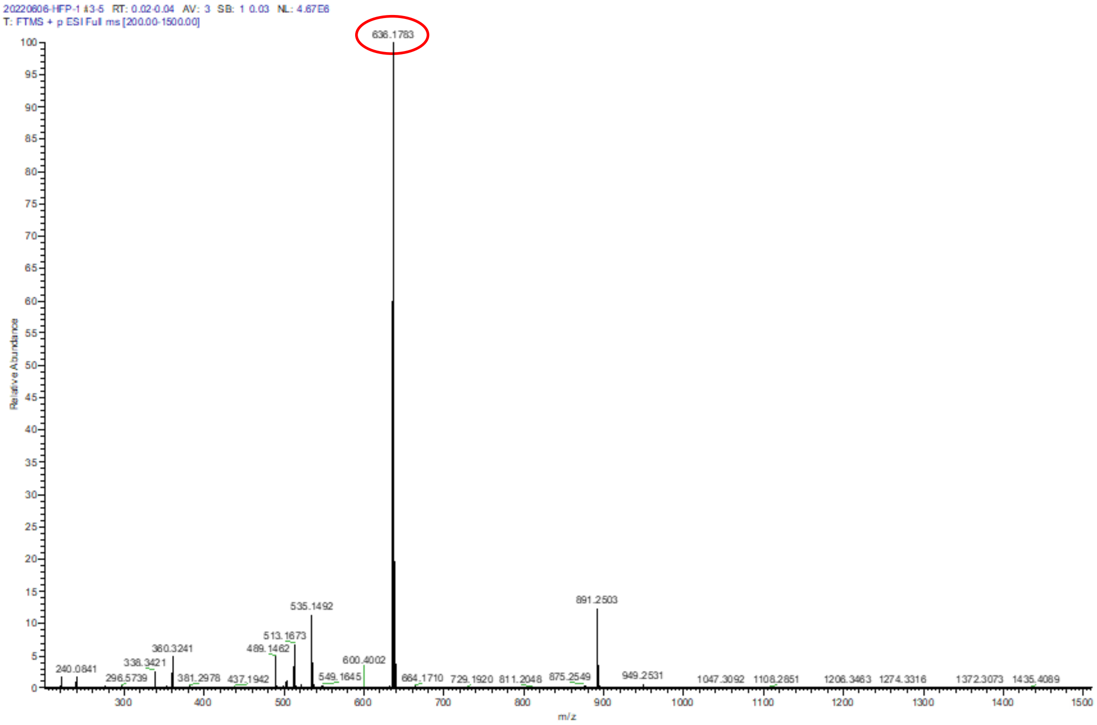

Figure S45. ESI-HRMS spectrum of **C2-NO$_2$**.

Compound **4**

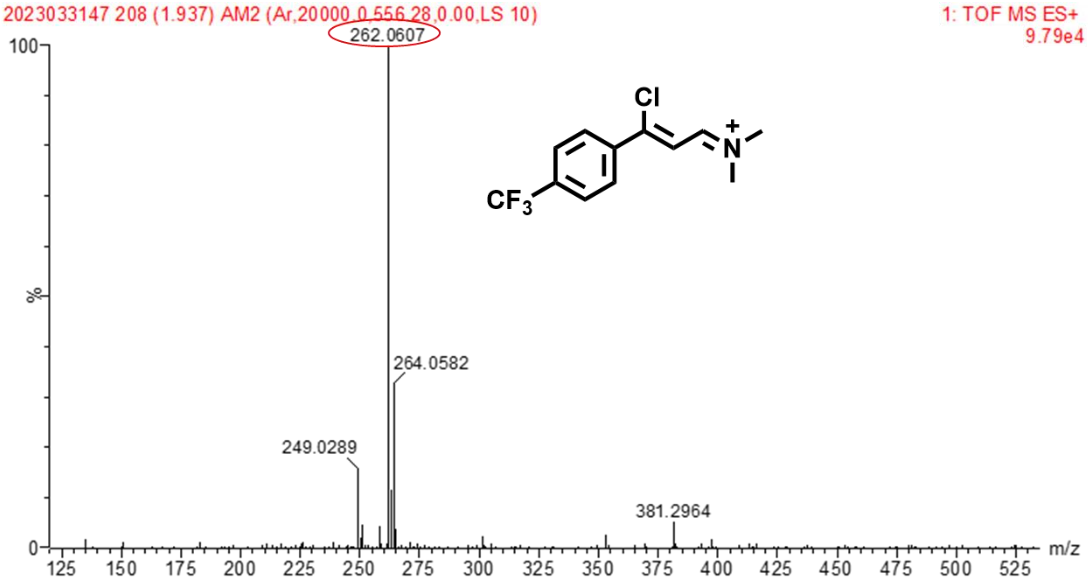

Figure S46. ESI-HRMS spectrum of Compound **4**.

C2-CF₃

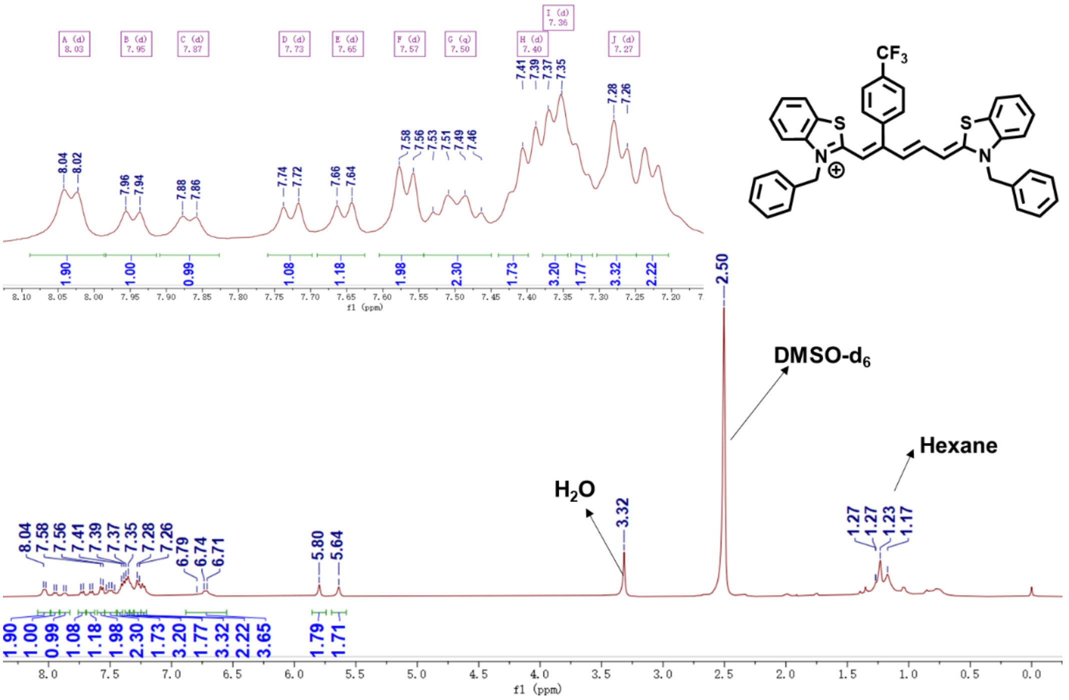

Figure S47. ¹H NMR spectrum of **C2-CF₃**.

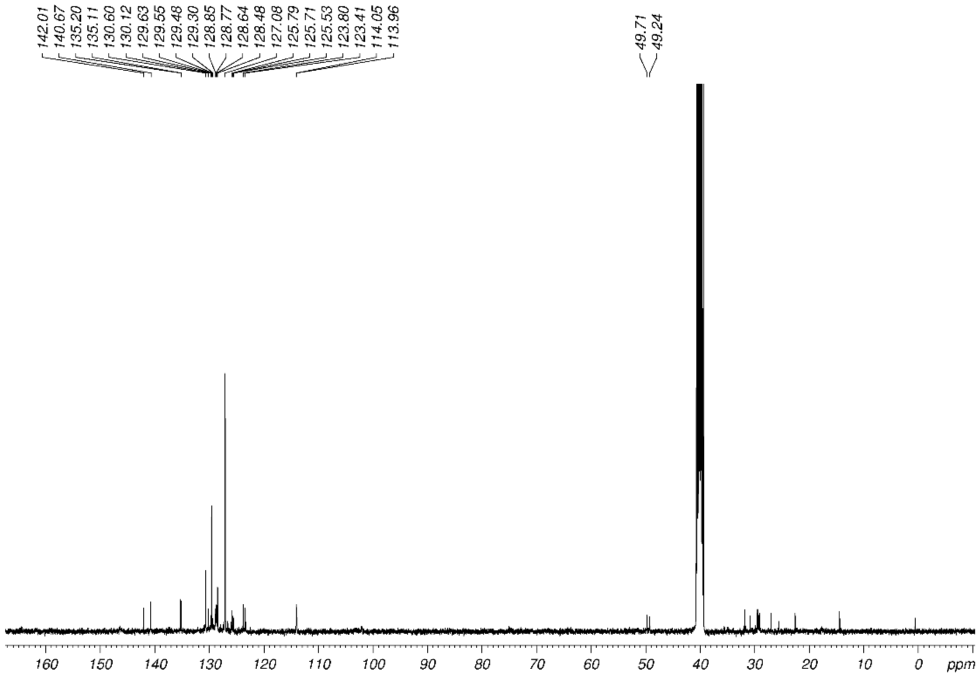

Figure S48. ^{13}C NMR spectrum of **C2-CF$_3$**.

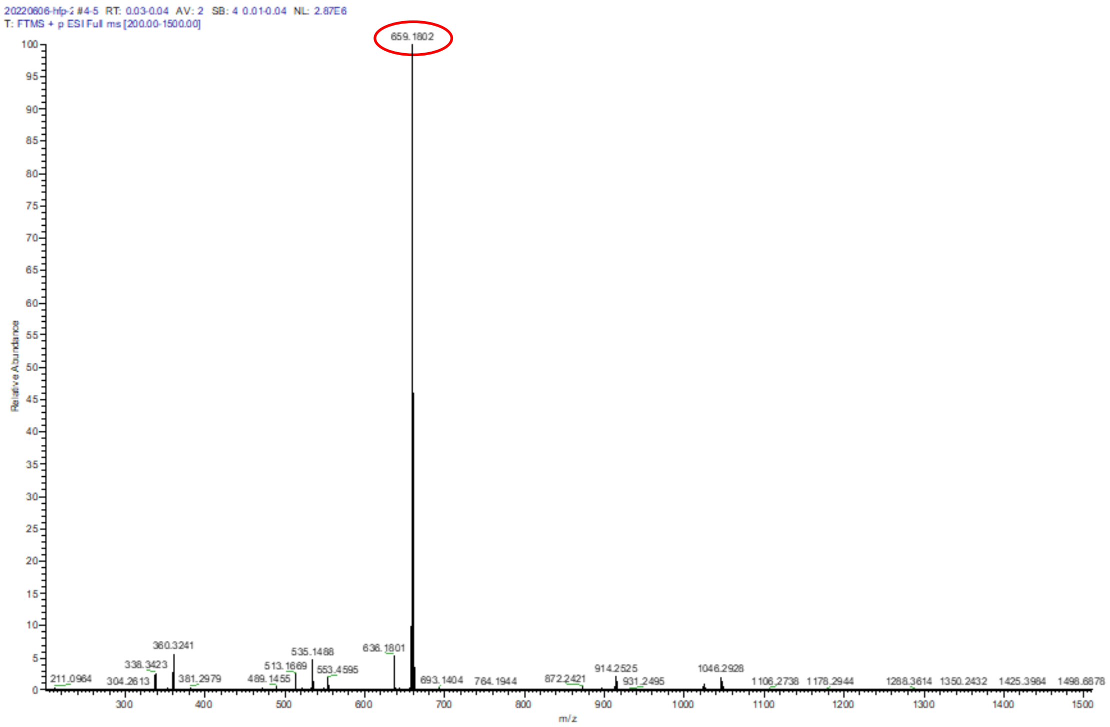

Figure S49. ESI-HRMS spectrum of **C2-CF$_3$**.

Compound 5

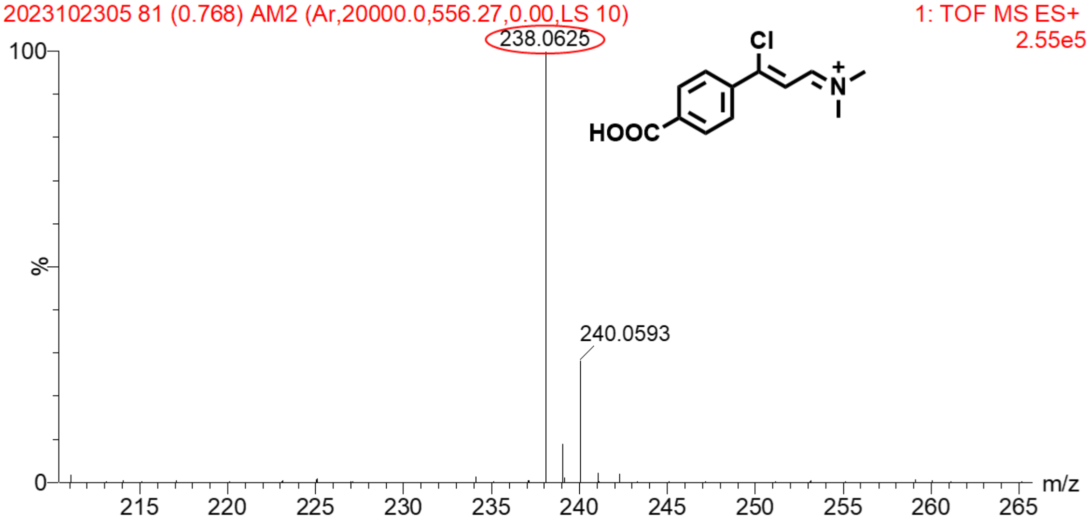

Figure S50. ESI-HRMS spectrum of Compound **5**.

C2-COOH

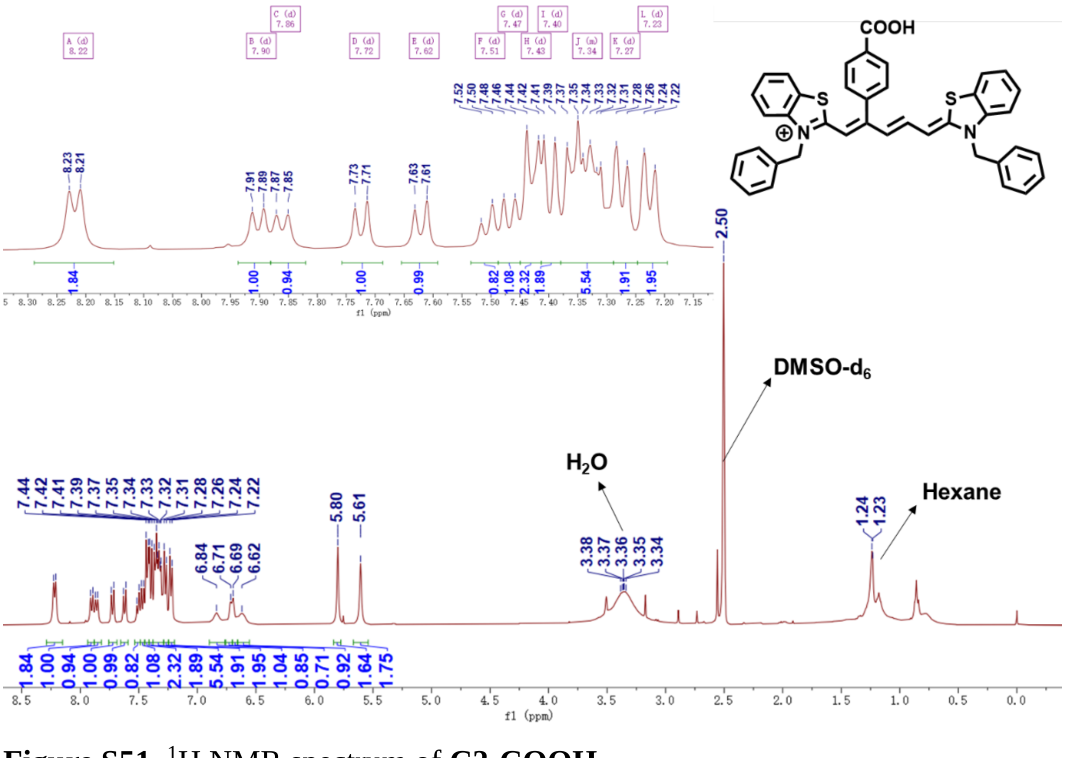

Figure S51. ^{1}H NMR spectrum of **C2-COOH**.

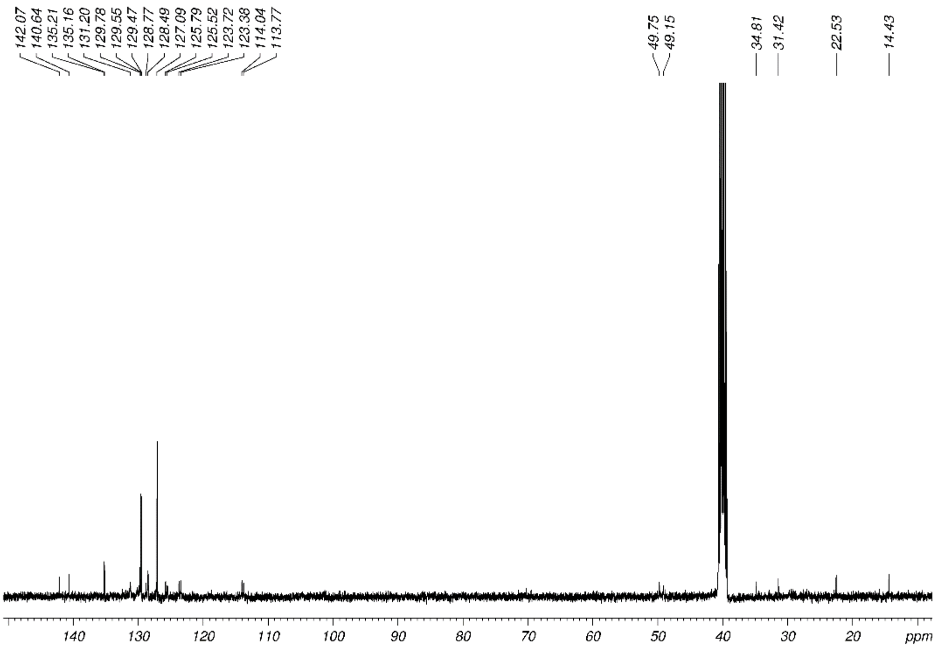

Figure S52. ^{13}C NMR spectrum of **C2-COOH**.

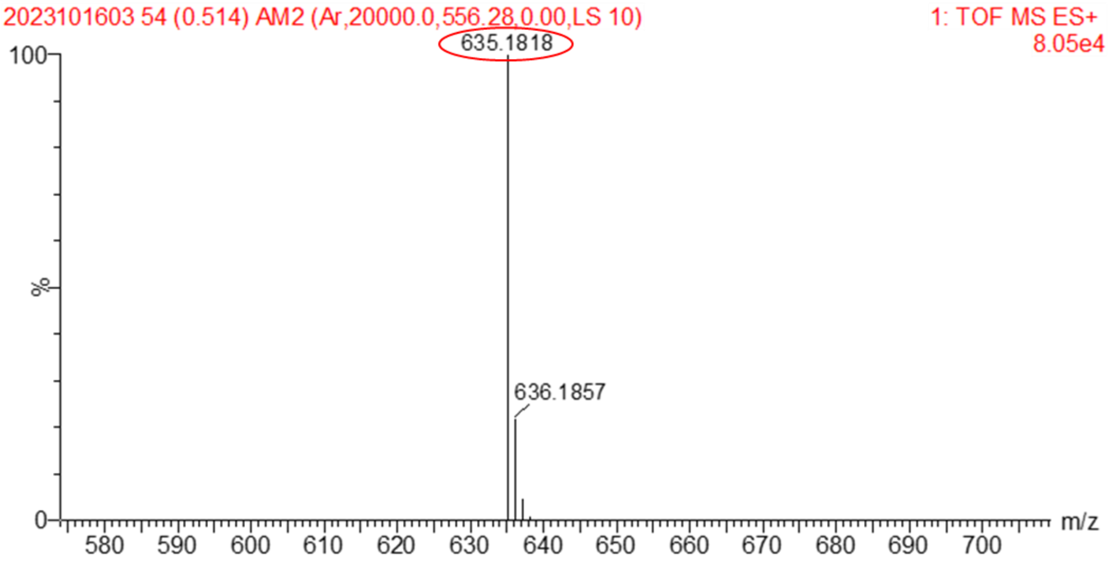

Figure S53. ESI-HRMS spectrum of **C2-COOH**.

Compound **6**

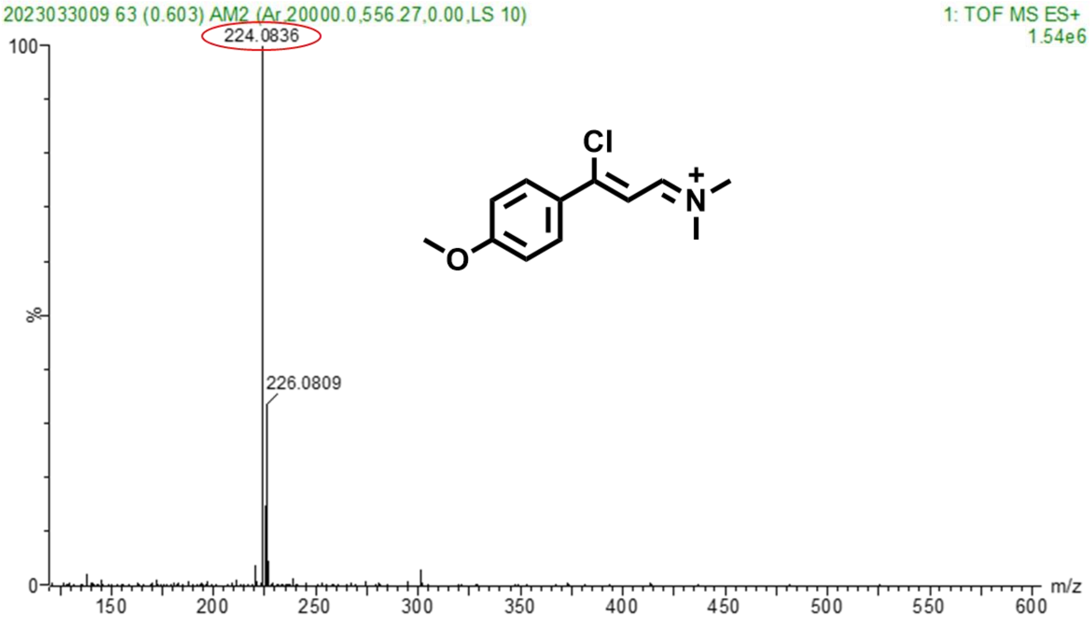

Figure S54. ESI-HRMS spectrum of Compound **6**.

C2-OMe

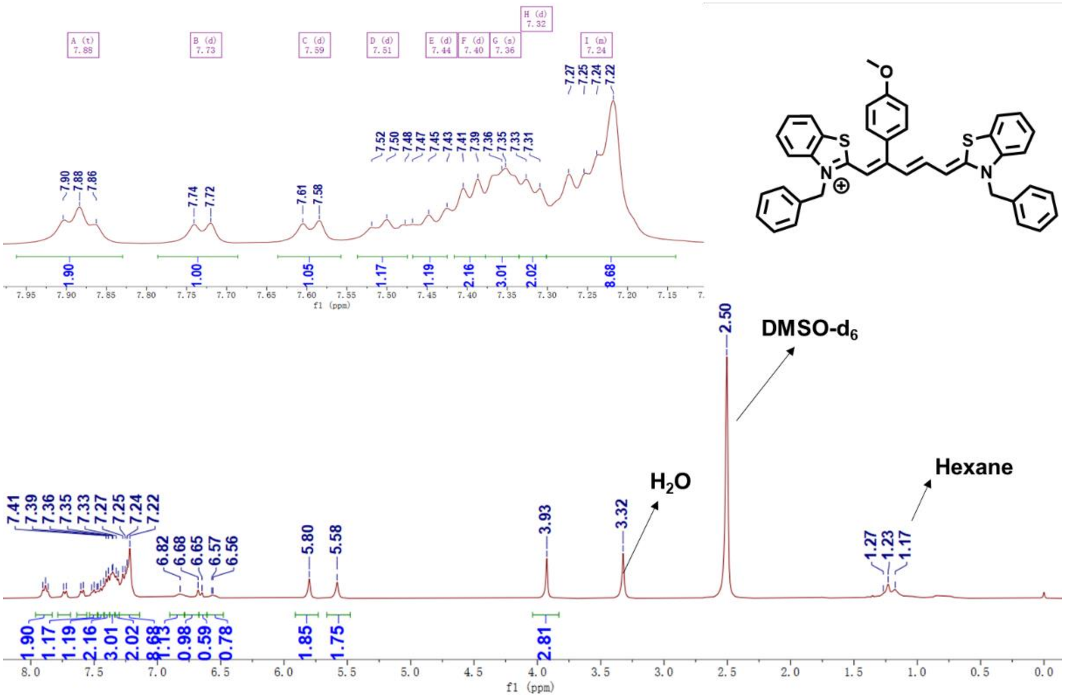

Figure S55. ^{1}H NMR spectrum of **C2-OMe**.

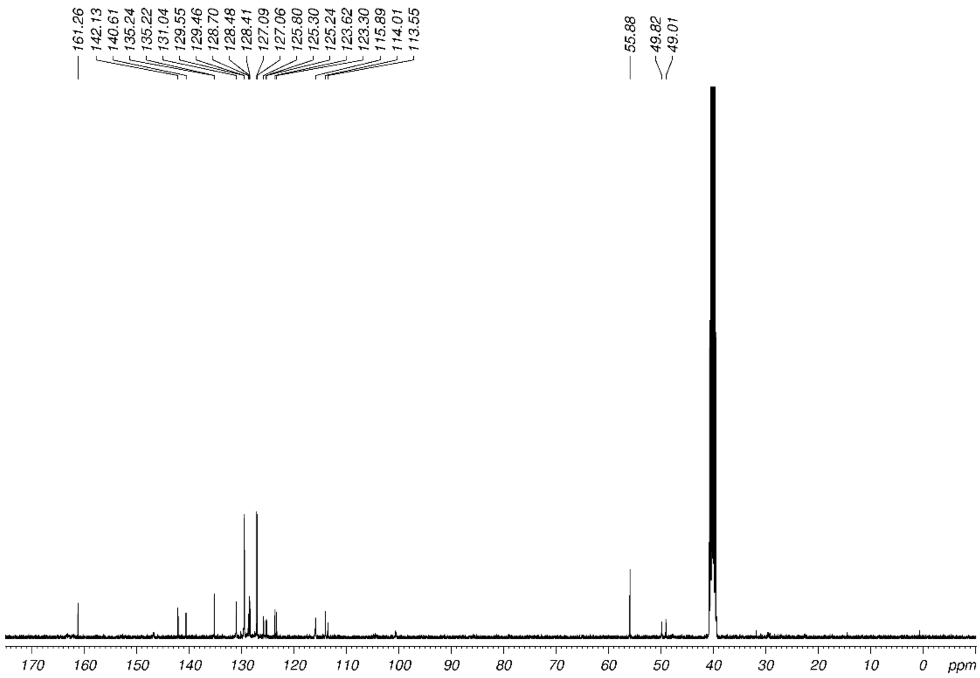

Figure S56. ^{13}C NMR spectrum of **C2-OMe**.

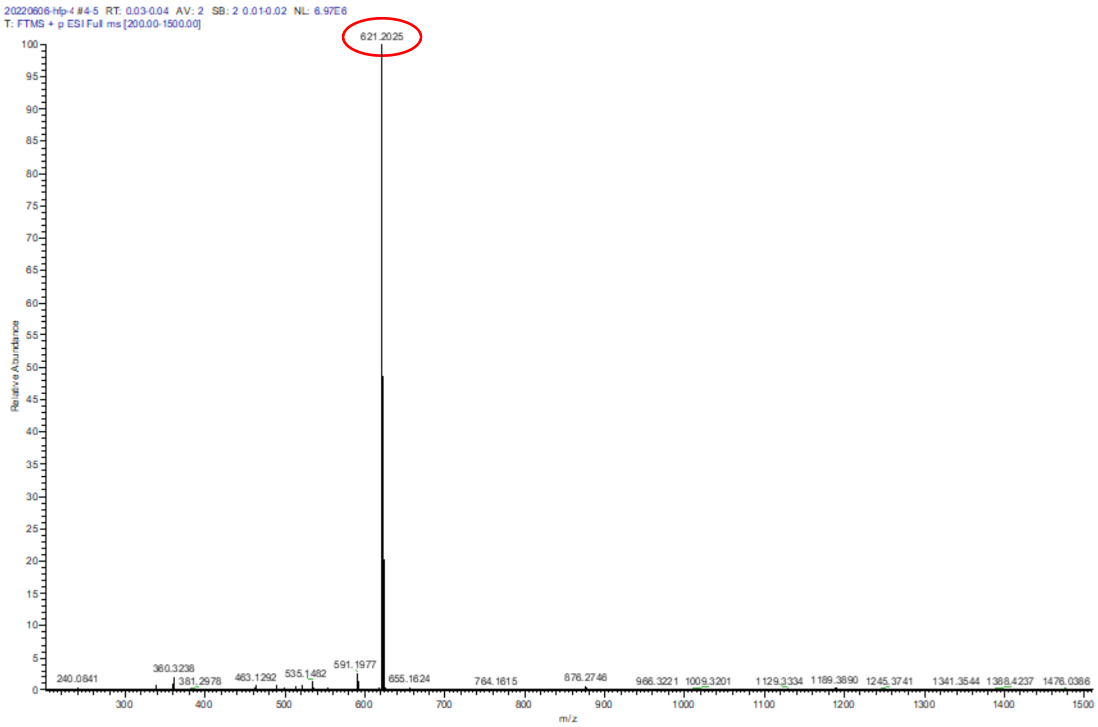

Figure S57. ESI-HRMS spectrum of **C2-OMe**.

Compound 7

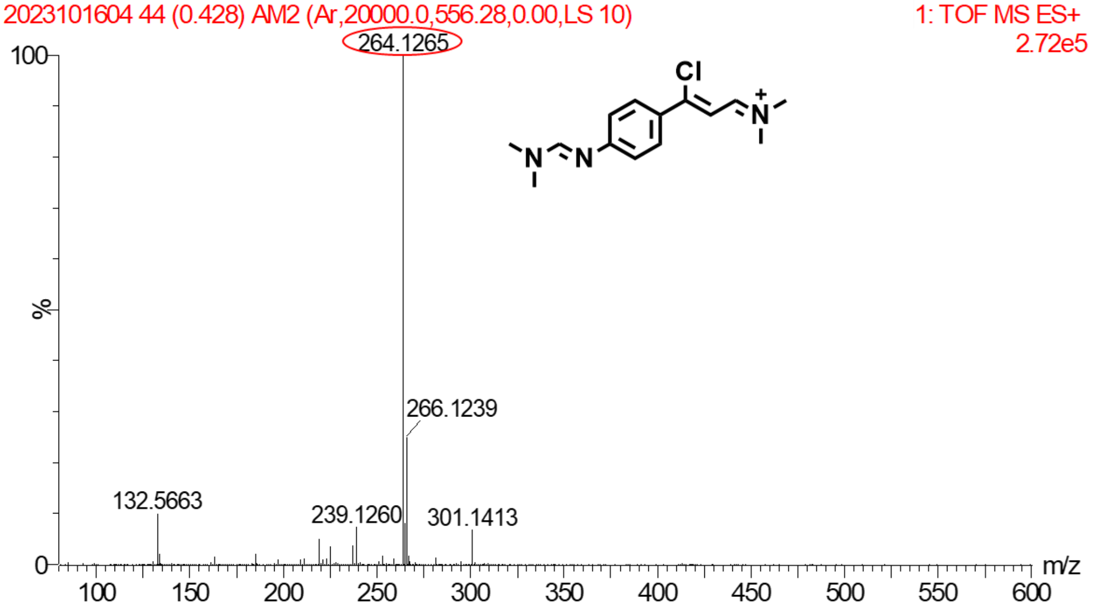

Figure S58. ESI-HRMS spectrum of **Compound 7**.

C2-NH2

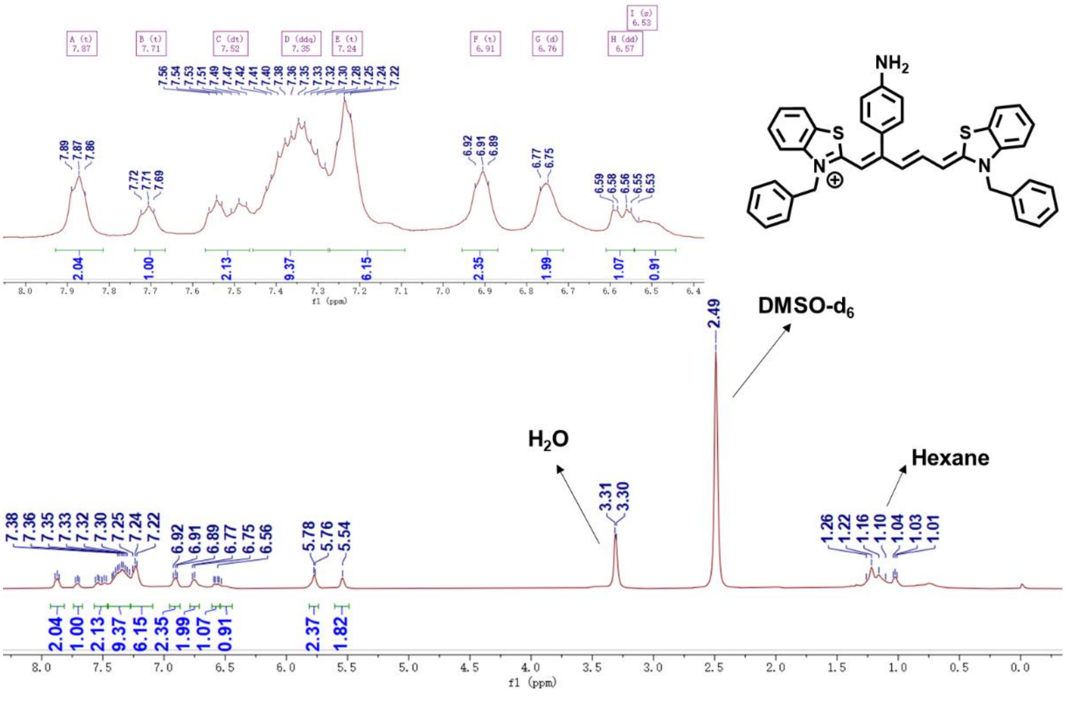

Figure S59. ^{1}H NMR spectrum of **C2-NH2**.

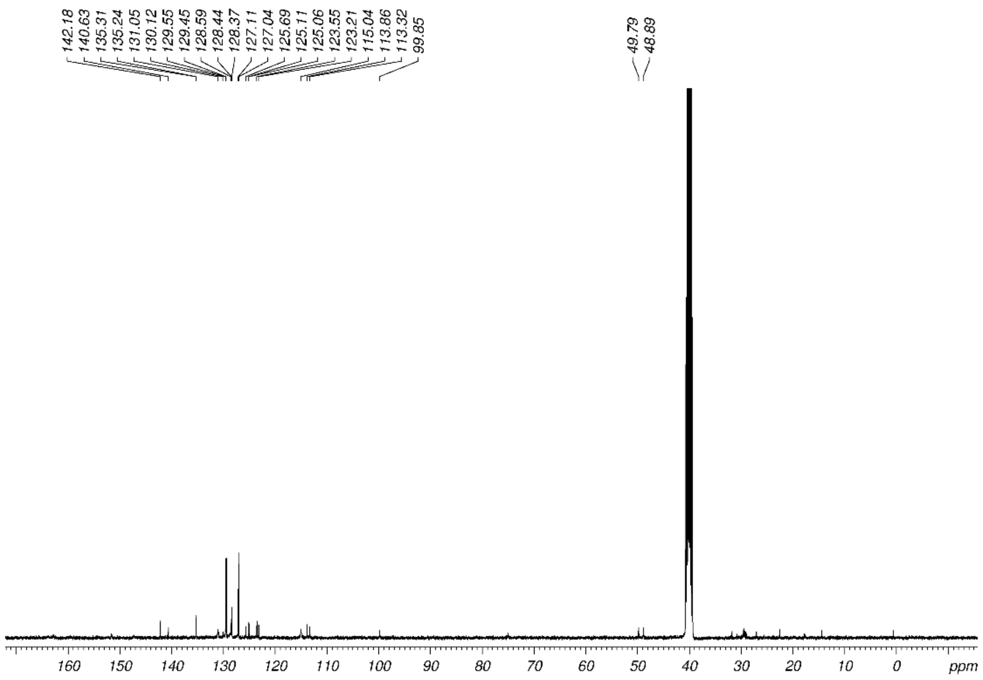

Figure S60. ^{13}C NMR spectrum of **C2-NH$_2$**.

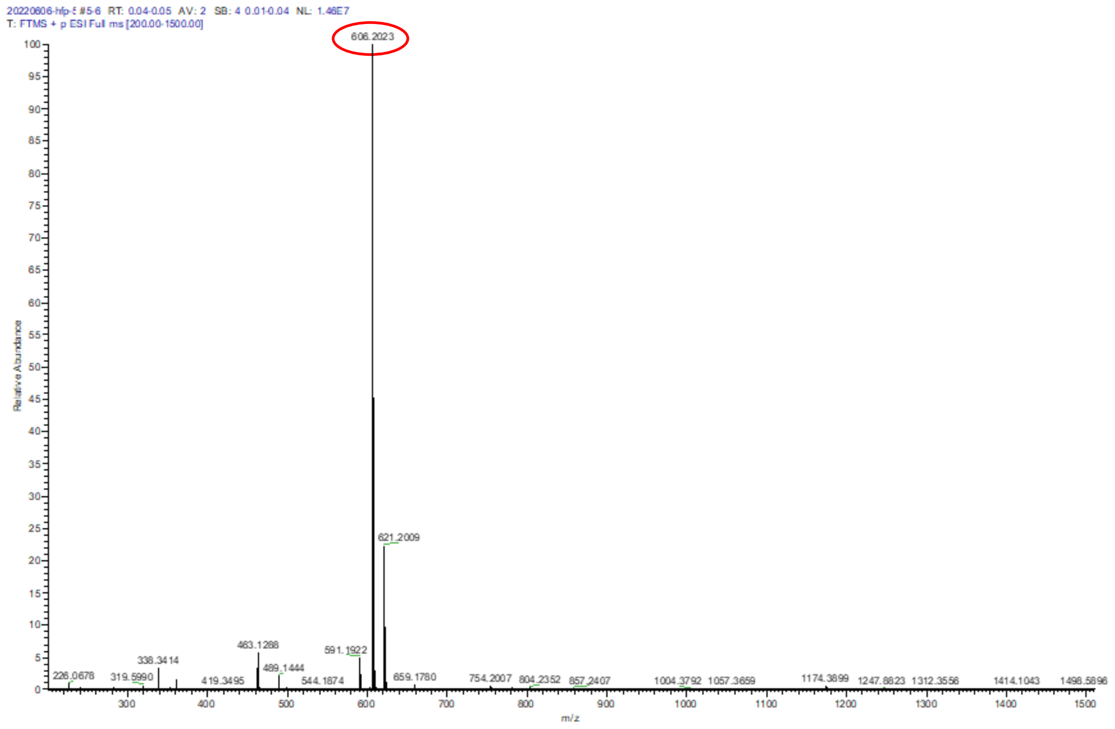

Figure S61. ESI-HRMS spectrum of **C2-NH$_2$**.

6. References

[1] Frisch, M. J.; Trucks, G. W.; Schlegel, H. B.; Scuseria, G. E.; Robb, M. A.; Cheeseman, J. R.; Scalmani, G.; Barone, V.; Petersson, G. A.; Nakatsuji, H.; Li, X.; Caricato, M.; Marenich, A. V.; Bloino, J.; Janesko, B. G.; Gomperts, R.; Mennucci, B.; Hratchian, H. P.; Ortiz, J. V.; Izmaylov, A. F.; Sonnenberg, J. L.; Williams; Ding, F.; Lipparini, F.; Egidi, F.; Goings, J.; Peng, B.; Petrone, A.; Henderson, T.; Ranasinghe, D.; Zakrzewski, V. G.; Gao, J.; Rega, N.; Zheng, G.; Liang, W.; Hada, M.; Ehara, M.; Toyota, K.; Fukuda, R.; Hasegawa, J.; Ishida, M.; Nakajima, T.; Honda, Y.; Kitao, O.; Nakai, H.; Vreven, T.; Throssell, K.; Montgomery Jr., J. A.; Peralta, J. E.; Ogliaro, F.; Bearpark, M. J.; Heyd, J. J.; Brothers, E. N.; Kudin, K. N.; Staroverov, V. N.; Keith, T. A.; Kobayashi, R.; Normand, J.; Raghavachari, K.; Rendell, A. P.; Burant, J. C.; Iyengar, S. S.; Tomasi, J.; Cossi, M.; Millam, J. M.; Klene, M.; Adamo, C.; Cammi, R.; Ochterski, J. W.; Martin, R. L.; Morokuma, K.; Farkas, O.; Foresman, J. B.; Fox, D. J. Gaussian 16 Rev. A.01, Wallingford, CT, **2016**.

[2] J.-D. Chai, M. Head-Gordon, *Phys. Chem. Chem. Phys.* **2008**, *10*, 6615-6620.

[3] A. V. Marenich, C. J. Cramer, D. G. Truhlar, *J. Phys. Chem. B* **2009**, *113*, 6378-6396.

[4] C. Wang, W. Chi, Q. Qiao, D. Tan, Z. Xu, X. Liu, *Chem. Soc. Rev.* **2021**, *50*, 12656-12678.

[5] W. Chi, J. Chen, W. Liu, C. Wang, Q. Qi, Q. Qiao, T. M. Tan, K. Xiong, X. Liu, K. Kang, Y.-T. Chang, Z. Xu, X. Liu, *J. Am. Chem. Soc.* **2020**, *142*, 6777-6785.

[6] F. Neese, *WIREs Comput. Mol. Sci.* **2022**, *12*, e1606.

[7] H. Ma, S. Long, J. Cao, F. Xu, P. Zhou, G. Zeng, X. Zhou, C. Shi, W. Sun, J. Du, K. Han, J. Fan, X. Peng, *Chem. Sci*. **2021**, 12, 13809-13816.
